# Neural mass modeling of slow-fast dynamics of seizure initiation and abortion

**DOI:** 10.1371/journal.pcbi.1008430

**Published:** 2020-11-09

**Authors:** Elif Köksal Ersöz, Julien Modolo, Fabrice Bartolomei, Fabrice Wendling

**Affiliations:** 1 University of Rennes, Inserm-U1099, LTSI, Rennes, France; 2 Aix Marseille University, Inserm, INS, Institut de Neurosciences des Systèmes, Marseille, France; 3 APHM, Timone Hospital, Clinical Neurophysiology, Marseille, France; Newcastle University, UNITED KINGDOM

## Abstract

Epilepsy is a dynamic and complex neurological disease affecting about 1% of the worldwide population, among which 30% of the patients are drug-resistant. Epilepsy is characterized by recurrent episodes of paroxysmal neural discharges (the so-called seizures), which manifest themselves through a large-amplitude rhythmic activity observed in depth-EEG recordings, in particular in local field potentials (LFPs). The signature characterizing the transition to seizures involves complex oscillatory patterns, which could serve as a marker to prevent seizure initiation by triggering appropriate therapeutic neurostimulation methods. To investigate such protocols, neurophysiological lumped-parameter models at the mesoscopic scale, namely neural mass models, are powerful tools that not only mimic the LFP signals but also give insights on the neural mechanisms related to different stages of seizures. Here, we analyze the multiple time-scale dynamics of a neural mass model and explain the underlying structure of the complex oscillations observed before seizure initiation. We investigate population-specific effects of the stimulation and the dependence of stimulation parameters on synaptic timescales. In particular, we show that intermediate stimulation frequencies (>20 Hz) can abort seizures if the timescale difference is pronounced. Those results have the potential in the design of therapeutic brain stimulation protocols based on the neurophysiological properties of tissue.

## Author summary

Epilepsy is a complex disease affecting 1% of the worldwide population of which 30% of the patients are drug-resistant and seeking for alternative therapeutic methods, such as neurostimulation. Epileptic seizures can be hallmarked by preceding pre-ictal phases which are a possible window of opportunity to trigger electrical stimulation with the objective to prevent seizure initiation. Biophysiological models are an appropriate framework to understand underlying dynamics and transitions between different epileptogenic phases. In this study, we consider a typical pre-ictal regime with complex bursting-type oscillations, which can be accurately reproduced by a neural mass model. By analyzing the multiple time-scaled structure of the model, we identify the key role of the subpopulations of GABAergic interneurons. We show that appropriate brain stimulation targeting GABAergic interneurons is able to abort pre-ictal bursting, thus preventing seizures to develop.

## Introduction

Epilepsy is a severe, multi-causal chronic disease defined by the recurrence of unpredictable seizures that severely affect patients’ quality of life. In 30% of patients, antiepileptic drugs [1] remain inefficient to control the occurrence of seizures. In most cases, drug-resistant epilepsies are ‘focal’ [2], as characterized by an epileptogenic zone (EZ) that is relatively circumscribed in one of the two cerebral hemispheres. There is a large body of evidence supporting that the balance between excitatory and inhibitory processes is modified in the EZ [3] due to multiple, not mutually exclusive, pathological mechanisms resulting from changes occurring at the cellular level (e.g. hyperexcitability caused by potassium and chloride dysregulation, review in [4]), up to the network level (e.g. hyperexcitability caused by altered glutamatergic or GABAergic synaptic transmission, review in [5]). Unfortunately, surgical treatment can only be offered to 15–20% drug-resistant patients [6] in whom the benefit-to-deficit ratio is favorable. Therefore, alternative therapeutic procedures aimed at reducing seizures’ frequency are urgently needed.

Among these procedures, direct electrical stimulation of the brain is an increasingly popular technique of treating epilepsy, as evidenced by both animal and human studies [7]. Stimulation targets have included deep brain structures such as thalamic nuclei, hippocampus or cortical targets [8]. It has been acknowledged for decades that stimulation of the cortex during routine brain mapping procedures may induce epileptiform discharges or seizures, but more recently pulse trains have demonstrated their potential in aborting abnormal epileptiform activity [9]. Direct stimulation has been shown to be effective in suppressing epileptic activity, however with inconsistent results among patients. Furthermore, brain stimulation in drug-refractory patients is recognized to be still largely empirical [10]. A rational definition of stimulation protocols is indeed still missing, as evidenced in randomized controlled trials [11].

In this context, the specific objective of the present study is to exploit neuro-inspired models to design neurostimulation protocols aiming at aborting seizures at their onset. More specifically, we investigate a well-defined pattern of interictal-to-ictal transition characterized by the occurrence of pre-ictal rhythmic large amplitude spikes followed by a fast onset activity, as observed in stereo-EEG recordings (SEEG, intracerebral electrodes). Although not the unique one, this commonly encountered pattern has long been considered as a hallmark of the EZ, especially in mesial temporal lobe epilepsy [12–14]. More particularly, we focus on pre-ictal bursting characterized by active episodes (fast epileptic spikes), repeated (quasi-) periodically and separated by quiescent (slow-wave and/or silent) phases. First, we accurately reproduce human electrophysiological patterns in a neural mass model (NMM) featuring glutamatergic pyramidal neurons as well as two types of GABAergic interneurons (somatostatin-positive or SOM+, and parvalbulmin-positive or PV+). After integrating neurostimulation effects in the model as a parametrizable exogenous membrane perturbation of the main cells and interneurons, we analyze the slow-fast nature of this nonlinear dynamical system in the bursting regime by using numerical bifurcation analysis and geometric singular perturbation theory (GSPT) [15,16]. Following this approach, the mechanisms leading to the pre-ictal bursting are determined, and the perturbation effects are explained geometrically. Here, the perturbation corresponds to a suprathreshold constant current stimulation that is stronger than that used for neuromodulation. In the following, the word "perturbation" refers to this "strong perturbation". Identification of the model structures to be targeted for bursting abortion highlight the key role of SOM+ interneurons in suppressing pre-ictal epileptic activity. Overall, the results of this methodological study elucidate mathematically the nature of pre-ictal spike bursting and provide aspiring information to design optimal direct stimulation protocols targeting this specific epileptiform pattern. To the best our knowledge, this is the first study deciphering explicitly the multiple time-scale structure of a neurophysiologically grounded NMM and modelling pre-ictal bursting.

## Model and methods

### Model

Since the early seventies, and following the pioneer works of W. Freeman [17] and F. Lopez da Silva [18], NMMs have been extensively used to study LFPs not only in the context of physiological activity (such as brain rhythms [19], and/or visually evoked potentials [20]), but also pathophysiological activity (epilepsy [21–24], Alzheimer’s disease [25], Parkinson’s disease [26]). NMMs are average descriptions of the temporal dynamics of neuronal assemblies, and therefore model parameters are themselves lumped. To some extent, these models are complementary of models of microcircuits in which neurons are explicitly represented. The important point there is that NMMs are neuro-inspired, since they implement interactions between different subpopulations of neurons (glutamatergic–excitatory- neurons and GABAergic–inhibitory- interneurons of various types) and model parameters are physiologically grounded [27,28]. Typically, the time constants governing average post-synaptic potentials (PSPs) match the rising and decaying times of corresponding experimentally recorded PSPs [27]. Regarding average firing rates, sigmoid functions account for threshold and saturation effects that are major features of neuronal physiology [28] and account for non-linearities in neuronal dynamics. Importantly, NMMs have been used to simulate LFPs which closely resemble those experimentally recorded, and led to predictions which could be experimentally verified [29].

We consider the NMM presented in [22] which includes three interacting neuronal subpopulations: pyramidal neurons (divided into two subpopulations) and inhibitory interneurons (SOM+ and PV+, also called “dendrite-projecting slow” and “soma-projecting fast” interneurons, respectively) (see **S1 Fig** for the block diagram of the model). The average PSP of each subpopulation is determined by two functions: 1) a ‘pulse-to-wave’ function, *S*(*v*) = 5/(1+exp(0.56(6−*v*))), transforming the incoming PSPs into a firing rate; and 2) the input firing rate is converted into the mean PSP of the corresponding subpopulation by a linear transformation, that is *h*(*t*) = *Wt*/*τ*_*w*_ exp(−*t*/*τ*_*w*_), where *W* represents the average synaptic gain and *τ*_*w*_ is the average synaptic time constant, which is related to rise and decay times of PSPs and lumps together the passive membrane time constant and all signal propagation delays. The system reads:

$$\begin{array}{l} {\ddot{y}}_{0}=\frac{A}{\tau_{a}}S(y_{1}-y_{2}-y_{3})-\frac{2}{\tau_{a}}{\dot{y}}_{0}-\frac{1}{\tau_{a}^{2}}y_{0},\\ {\ddot{y}}_{1}=\frac{A}{\tau_{a}}\{p(t)+C_{2}S(C_{1}y_{0})\}-\frac{2}{\tau_{a}}{\dot{y}}_{1}-\frac{1}{\tau_{a}^{2}}y_{1},\\ {\ddot{y}}_{2}=\frac{B}{\tau_{b}}C_{4}S(C_{3}y_{0})-\frac{2}{\tau_{b}}{\dot{y}}_{2}-\frac{1}{\tau_{b}^{2}}y_{2},\\ {\ddot{y}}_{3}=\frac{G}{\tau_{g}}C_{7}S(C_{5}y_{0}-{C_{6}y}_{4})-\frac{2}{\tau_{g}}{\dot{y}}_{3}-\frac{1}{\tau_{b}^{2}}y_{3}\\ {\ddot{y}}_{4}=\frac{B}{\tau_{b}}S(C_{3}y_{0})-\frac{2}{\tau_{b}}{\dot{y}}_{4}-\frac{1}{\tau_{b}^{2}}y_{4}. \end{array}$$ (1)

Variables *y*_*i*_ stand for the PSPs generated at the level of pyramidal cells (*y*_0_), excitatory inputs on pyramidal cells (*y*_1_) (or in other words, the pyramidal subpopulation denoted by y_0_ excites the pyramidal subpopulation y_1_), SOM+ interneurons (*y*_2_), PV+ interneurons (*y*_3_), and inhibitory inputs on PV+ interneurons (*y*_4_). Parameters *A*, *B*, *G* are the synaptic gains, *τ*_*a*_, *τ*_*b*_, *τ*_*g*_ are the synaptic time constants, connectivity constants *C*_*i*_s represent the average number of synaptic contacts, and *p*(*t*) is the external (noisy) cortical input *p*(*t*) = *p*+*ξ*, where *p* is the mean of the external input, and *ξ* is a random variable following a normal distribution with zero mean and standard deviation *σ*). Table 1 presents the parameter values used in this manuscript unless otherwise stated. The main difference between the parameter sets of [22] and Table 1 is the connectivity strengths of the circuit involving PV+ interneurons. Note that *τ*_*a*_ is 3.3 times and *τ*_*b*_ is 16.6 times greater than *τ*_*g*_. These differences would introduce multiple time-scale dynamics in the system. Below, we recall primaries of slow-fast analysis before expressing (1) in slow-fast formulation.

**Table 1:** Parameter values during simulated background activity.

*A* (mV)	*B* (mV)	*G* (mV)	*p* (Hz)	*C*_1_	*C*_2_	*C*_3_	*C*_4_	*C*_5_	*C*_6_	*C*_7_	*τ*_*a*_(s)	*τ*_*b*_(*s*)	*τ*_*g*_(s)
5	40	35	90	135	108	35	25	450	121	121	0.01	0.05	0.003

### Primaries of slow-fast analysis

Complex oscillations, e.g. spiking, bursting and mixed-mode oscillations, are widely present in electrophysiological signals, and are hallmarked by interacting components running in different timescales (for a recent review see [30]). Mathematical models describing these dynamics involve inevitably variables with different timescales. For example, the interaction of two variables running at two different time-scales (1 fast and 1 slow variables) is sufficient for generating spiking, whereas bursting emerges only in higher dimensional systems with at least two different time-scales (2 fast and 1 slow variables/subsystems). Geometric methods in the singular limits (denoted as slow-fast or multiple timescale analysis) simplify the investigation of the model dynamics by breaking into several reduced systems. For instance, bursting patterns can be classified in a singular limit where the slowest variables are treated as parameters [31,32].

A slow-fast system in the general slow form reads,

$$\begin{array}{c} \epsilon \dot{x}=f(x,z,\epsilon ),\\ \dot{z}=g(x,z,\epsilon ), \end{array}$$

with fast variables *x* and slow variables *z* of arbitrary dimensions, time scale parameter 0<*ϵ*≪1, and dot represents derivation with respect to time *t*. The dynamics of a slow-fast system can be divided into *fast* and *slow* epochs. Each of these epochs can be investigated with the slow-fast analysis in a hybrid manner and then can be concatenated, so that one can understand the underlying structure giving sharp transitions (excitable responses to external inputs) and complex oscillatory patterns (spiking, bursting and subthreshold oscillations) [33].

An important geometrical object for both the slow and the fast dynamics is the *critical manifold* $C^{0}$, defined as the nullcline of the fast variable $C^{0}=\{\left(x,z\right))|f(x,z,0)=0\}$, eventually obtained by setting *ε* = 0. For the differential-algebraic system defined for *ϵ* = 0, the so-called *reduced system (slow subsystem)* approximates the slow dynamics of the original system. The critical manifold $C^{0}$ both defines the phase space of the reduced system and equilibrium points of the layer problem expressed in the fast time-scale, that is

$$\begin{array}{c} x\prime =f(x,z,0),\\ z^{\prime }=0, \end{array}$$

where (′) denotes derivative with respect to *τ* = *t*/*ϵ*. The stability of the layer problem determines the characteristics of the critical manifold. The critical manifold $C^{0}$ is normally hyperbolic along the set for which det(*f*_*x*_(*x*,*z*,0))≠0, which can be attracting, repelling or saddle type. The Fenichel theory [15] guarantees that these normally hyperbolic points of the critical manifold perturb smoothly in *ϵ* and give *slow manifolds* ($C^{\epsilon }$) of the original system for small enough *ϵ*>0. If C is folded, attracting and repelling branches of *C*^0^ meet along the fold set ${F=\{det(f}_{x}(x_{fold},z_{fold},0))=0\}$, where normal hyperbolicity is lost. Extension of the classical Fenichel theory to non-hyperbolic sets provides a tool to investigate the slow dynamics near F, and one can expect *canard solutions* in the neighborhood of such sets [34].

### Slow-fast formulation of the model

One can notice that the variable *y*_4_ in (1) is equivalent to *y*_2_, thus the dimension of (1) can be reduced by multiplying the PSP variables with *C*_*i*_s before the ‘pulse-to-wave’ conversion. Further, by applying the variable conversion,

$$\left(\frac{y_{0}}{\tau_{a}},\frac{y_{1}}{\tau_{a}},\frac{y_{2}}{\tau_{b}},\frac{y_{3}}{\tau_{g}},y_{5},y_{6},y_{7},y_{8}\right)\to (v_{0},v_{1},v_{2},v_{3},v_{4},y_{5},y_{6},y_{7},y_{8}),$$

system (1) can be written as:

$$\begin{array}{l} \tau_{g}{\dot{v}}_{3}=y_{8},\\ \tau_{g}\dot{y_{8}}=G\;S(C_{5}\tau_{a}v_{0}-C_{6}\tau_{b}v_{2})-v_{3}-2y_{8},\\ \tau_{a}{\dot{v}}_{0}=y_{5},\\ \tau_{a}\dot{y_{5}}=A\;S({A\tau_{a}p+C_{2}\tau_{a}v_{1}-C}_{4}\tau_{b}v_{2}-C_{7}\tau_{g}v_{3})-v_{0}-2y_{5},\\ \tau_{a}{\dot{v}}_{1}=y_{6},\\ \tau_{a}\dot{y_{6}}=A\;S(C_{1}\tau_{a}v_{0})-v_{1}-2y_{6},\\ \tau_{b}{\dot{v}}_{2}=y_{7},\\ \tau_{b}\dot{y_{7}}=B\;S(C_{3}\tau_{a}v_{0})-v_{2}-2y_{7}. \end{array}$$ (2)

Intuitively, system (1), hence system (2), are multiple-time-scale systems which can result in complex epileptogenic patterns for appropriate choices of parameters. Thus, understanding the multiple-time-scale structure of (2) is indispensable for designing brain stimulation protocols aiming at aborting the aforementioned oscillatory patterns. In order to proceed a slow-fast analysis of (2), where the time-scaling in not explicit, and use the standard methods of GSPT, we first normalize time *t* with respect to *τ*_*g*_, as $\tilde{t}=t/\tau_{g}$, then define two parameters, *δ* = *τ*_*g*_/*τ*_*a*_ and *ε* = *τ*_*a*_/*τ*_*b*_. We further assume that *ε* and *δ* are independent of the synaptic time constants (*τ*_*a*_,*τ*_*b*_,*τ*_*g*_), similar to the approach followed in [35], that is to say variations in (*ε*,*δ*) for theoretical analysis do not require varying (*τ*_*a*_,*τ*_*b*_,*τ*_*g*_). At the end system (2) is expressed in an explicit slow-fast formulation:

$$\begin{array}{l} \frac{{dv}_{3}}{d\tilde{t}}=y_{8}≔F_{3}(y_{8}),\\ \frac{{dy}_{8}}{d\tilde{t}}=G\;S(C_{5}\tau_{a}v_{0}-C_{6}\tau_{b}v_{2})-v_{3}-2y_{8}≔F_{8}(v_{0},v_{2},v_{3},y_{8}),\\ \frac{dv_{0}}{d\tilde{t}}=\delta y_{5}≔{\delta F}_{0}(y_{5}),\\ \frac{dy_{5}}{d\tilde{t}}=\delta (A\;S({A\tau_{a}p+C_{2}\tau_{a}v_{1}-C}_{4}\tau_{b}v_{2}-C_{7}\tau_{g}v_{3})-v_{0}-2y_{5})≔F_{5}(v_{0},v_{1},v_{2},v_{3},y_{5}),\\ \frac{dv_{1}}{d\tilde{t}}=\delta y_{6}≔{\delta F}_{1}(y_{6}),\\ \frac{dy_{6}}{d\tilde{t}}=\delta (A\;S(C_{1}\tau_{a}v_{0})-v_{1}-2y_{6})≔{\delta F}_{6}(v_{0},v_{1},y_{6}),\\ \frac{dv_{2}}{d\tilde{t}}=\delta \varepsilon y_{7}≔{\delta \varepsilon F}_{2}(y_{7}),\\ \frac{dy_{7}}{d\tilde{t}}=\delta \varepsilon (B\;S(C_{3}\tau_{a}v_{0})-v_{2}-2y_{7})≔{\delta \varepsilon F}_{7}(v_{0},v_{2},y_{7}). \end{array}$$ (3)

System (3) is a three-time-scale system for small enough values of (*δ*,*ε*) [27–31], with (*v*_3_, *y*_8_) being fast variables, (*v*_0_, *y*_5_, *v*_1_, *y*_6_) slow variables, and (*v*_2_, *y*_7_) superslow variables. System (3) is written using the (fast) time $\tilde{t}$, and called the *fast system*. As can be noticed, rescaling of (1) identifies the small parameters such that GSPT can be applied and expresses the importance of timescales. We follow [36,37] to analyze the three time scaled slow-fast structure of (3). Defining ${\tilde{t}}_{s}=\delta \tilde{t}$ gives the *slow system*:

$$\begin{array}{l} \;\delta \frac{{dv}_{3}}{d{\tilde{t}}_{s}}=F_{3}(y_{8})\\ \delta \frac{{dy}_{8}}{d{\tilde{t}}_{s}}=F_{8}(v_{0},v_{2},v_{3},y_{8}),\\ \frac{dv_{0}}{d{\tilde{t}}_{s}}={F_{0}(y}_{5}),\\ \frac{dy_{5}}{d{\tilde{t}}_{s}}=F_{5}(v_{0},v_{1},v_{2},v_{3},y_{5}),\\ \frac{dv_{1}}{d{\tilde{t}}_{s}}=F_{1}(y_{6}),\\ \frac{dy_{6}}{d{\tilde{t}}_{s}}=F_{6}(v_{0},v_{1},y_{6}),\\ \frac{dv_{2}}{d{\tilde{t}}_{s}}={\varepsilon F}_{2}(y_{7}),\\ \frac{dy_{7}}{d{\tilde{t}}_{s}}=\varepsilon F_{7}(v_{0},v_{2},y_{7}). \end{array}$$ (4)

where *F*_*i*_s are as defined for (3), with *i* representing the system variables’ indices on the left-hand side. **S2A Fig** presents the bifurcation diagram of the (*v*_3_, *y*_8_, *v*_0_, *y*_5_, *v*_1_, *v*_6_)-subsystem of (4) for *ε* = 0, where *v*_2_ acts as a parameter, and a periodic bursting orbit of (3) for *ε* = 0.01. We see that the orbit agrees with the bifurcation diagram when *ε* is decreased. Details of the bursting behavior are explained in Sec. *Bursting analysis*.

Defining ${\tilde{t}}_{ss}=\varepsilon {\tilde{t}}_{s}=\varepsilon \delta \tilde{t}$ gives *the superslow system*,

$$\begin{array}{l} \varepsilon \delta \frac{dv_{3}}{d{\tilde{t}}_{ss}}=F_{3}(y_{8}),\\ \varepsilon \delta \frac{dy_{8}}{d{\tilde{t}}_{ss}}=F_{8}\left(v_{0},v_{2},v_{3},y_{8}\right),\\ \varepsilon \frac{dv_{0}}{d{\tilde{t}}_{ss}}=F_{0}\left(y_{5}\right),\\ \varepsilon \frac{dy_{5}}{d{\tilde{t}}_{ss}}=F_{5}\left(v_{0},v_{1},v_{2},v_{3},y_{5}\right),\\ \varepsilon \frac{dv_{1}}{d{\tilde{t}}_{ss}}=F_{1}\left(y_{6}\right),\\ \varepsilon \frac{dy_{6}}{d{\tilde{t}}_{ss}}=F_{6}\left(v_{0},v_{1},y_{6}\right),\\ \frac{dv_{2}}{d{\tilde{t}}_{ss}}=F_{2}\left(y_{7}\right),\\ \frac{dy_{7}}{d{\tilde{t}}_{ss}}=F_{7}\left(v_{0},v_{1},y_{7}\right). \end{array}$$ (5)

Systems (3), (4) and (5) are equivalent if *ε*≠0 and *δ*≠0, but they give nonequivalent dynamics in the singular limits *ε*→0 and/or *δ*→0. The limit *δ*→0 in the fast system (3) eliminates the slow and superslow dynamics and yields the *fast layer problem*,

$$\begin{array}{l} \frac{{dv}_{3}}{d\tilde{t}}=F_{3}(y_{8}),\\ \frac{{dy}_{8}}{d\tilde{t}}=F_{8}(v_{0},v_{2},v_{3},y_{8}), \end{array}$$ (6)

which describes the dynamics of the fast variables (*v*_3_, *y*_8_) for fixed values of (*v*_0_, *v*_2_), $\left(v_{0}^{0},v_{2}^{0}\right)$ for instance. The critical manifold is defined by the four-dimensional set of equilibria of the fast layer problem (6), which reads,

$$S^{0}=\{(v_{3},y_{8},v_{0},v_{2})|F_{3}(y_{8})=0\cap F_{8}(v_{0},v_{2},v_{3},y_{8})=0\},$$

and *S*^0^ is eventually in the (*y*_8_ = 0)-space. The stability of *S*^0^ is determined by deriving the Jacobian of *S*^0^ with respect to the fast variables, that is,

$$\mathrm{Jac}(S_{v_{3,y_{8}}}^{0})=[\begin{array}{cc} 0 & 1\\ -1 & -2 \end{array}].$$

Since $\det\left(Jac(S_{v_{3,y_{8}}}^{0})\right)\neq 0$, and the eigenvalues are *λ*_1,2_ = −1, the *S*^0^ is normally hyperbolic and stable. Hence, *S*^0^ is perturbed to local invariant slow manifolds for sufficiently small *δ*>0.

Another singular limit is obtained by letting *δ*→0 in the slow system (4) gives the algebraic-differential *slow reduced problem*,

$$\begin{array}{l} 0=F_{3}(y_{8}),\\ 0=F_{8}(v_{0},v_{2},v_{3},y_{8}),\\ \frac{dv_{0}}{d{\tilde{t}}_{s}}={F_{0}(y}_{5}),\\ \frac{dy_{5}}{d{\tilde{t}}_{s}}=F_{5}(v_{0},v_{1},v_{2},v_{3},y_{5}),\\ \frac{dv_{1}}{d{\tilde{t}}_{s}}=F_{1}(y_{6}),\\ \frac{dy_{6}}{d{\tilde{t}}_{s}}=F_{6}(v_{0},v_{1},y_{6}),\\ \frac{dv_{2}}{d{\tilde{t}}_{s}}={\varepsilon F}_{2}(y_{7}),\\ \frac{dy_{7}}{d{\tilde{t}}_{s}}=\varepsilon F_{7}(v_{0},v_{2},y_{7}), \end{array}$$ (7)

which describes the dynamics on *S*^0^. System (7) is a two-time-scale problem for *ε* sufficiently small and it gives the *slow layer problem* in the *ε*→0 limit,

$$\begin{array}{l} 0=F_{3}(y_{8}),\\ 0=F_{8}(v_{0},v_{2},v_{3},y_{8}),\\ \frac{dv_{0}}{d{\tilde{t}}_{s}}={F_{0}(y}_{5}),\\ \frac{dy_{5}}{d{\tilde{t}}_{s}}=F_{5}(v_{0},v_{1},v_{2},v_{3},y_{5}),\\ \frac{dv_{1}}{d{\tilde{t}}_{s}}=F_{1}(y_{6}),\\ \frac{dy_{6}}{d{\tilde{t}}_{s}}=F_{6}(v_{0},v_{1},y_{6}), \end{array}$$ (8)

where *v*_2_ appears as a parameter. A periodic orbit of (7) for *ε* = 0.01 and the bifurcation diagram of (8) as a function of *v*_2_ is projected on the (*v*_0_, *v*_2_)-plane in **S2B Fig**.

In the slow layer problem (8), the slow variables (*v*_0_, *y*_5_, *v*_1_, *y*_6_) evolve along fibers defined by ${(v_{3},y_{8},v}_{0},y_{5},v_{1},y_{6}{,v}_{2},y_{7})={(v_{3},y_{8},v}_{0},y_{5},v_{1},y_{6,}v_{2}^{0},y_{7}^{0}),$ where $\left(v_{2}^{0},y_{7}^{0}\right)$ are constant and ${(v_{3},y_{8},v}_{0},y_{5},v_{1},y_{6}v_{2}^{0},y_{7}^{0})$ restricted to *S*^0^. The equilibria of (8) defines the *superslow manifold L*^0^

$$L^{0}=\{{(v_{3},y_{8},v}_{0},y_{5},v_{1},y_{6}v_{2},y_{7})\in S^{0}|\begin{array}{c} F_{3}(y_{8})=0\cap F_{8}(v_{0},v_{2},v_{3},y_{8})=0\cap \\ {F_{0}(y}_{5})=0\cap F_{5}(v_{0},v_{1},v_{2},v_{3},y_{5})=0\cap \\ F_{1}(y_{6})=0\cap F_{6}(v_{0},v_{1},y_{6})=0 \end{array}\},$$

which is a subset of *S*^0^. The superslow manifold *L*^0^ is reduced to

$$\begin{array}{c} L^{0}=\{{(v_{3},y_{8},v}_{0},y_{5},v_{1},y_{6}v_{2},y_{7})\in S^{0}|\\ A\;S({A\tau_{a}p+C_{2}\tau_{a}AS(C_{1}\tau_{a}v_{0})-C}_{4}\tau_{b}v_{2}-C_{7}\tau_{g}v_{3})-v_{0}=0\}, \end{array}$$

where *v*_3_ = *G S*(*C*_5_*τ*_*a*_*v*_0_−*C*_6_*τ*_*b*_*v*_2_) is on *S*^0^. The curve *L*^0^ perturbs to locally slow invariant manifolds for *ε*>0 along the hyperbolic branches of *L*^0^, while the dynamics of near the non-hyperbolic fold points should be investigated using GSPT. Finally, the *superslow reduced problem* obtained by setting *ε*→0 in (5) reads

$$\begin{array}{l} \frac{dv_{2}}{d{\tilde{t}}_{s}}=F_{2}(y_{7}),\\ \frac{dy_{7}}{d{\tilde{t}}_{s}}=F_{7}(v_{0},v_{2},y_{7}). \end{array}$$ (9)

This algebraic-differential system determines the superslow dynamics restricted to *L*^0^, and eventually to *S*^0^.

### Strongly perturbed system

Electrical (through direct stimulation) and optical (through optogenetics, using light pulses in genetically modified animals) perturbations can alter action potential firing through modification of the mean membrane potential of the considered neural subpopulation. In the following, we use the word “perturbation” to refer to a strong suprathreshold input. We assumed an additive model for the stimulation effect onto the mean membrane PSP [38]. Thus, the external input *I*_*ext*_(*t*) is included in the ‘pulse-to-wave’ functions of the NMM in (3), and the system reads:

$$\begin{array}{l} \tau_{g}{\dot{v}}_{3}=y_{8},\\ \tau_{g}\dot{y_{8}}=G\;S(C_{5}\tau_{a}v_{0}-C_{6}\tau_{b}v_{2}+k_{PV}I_{ext})-v_{3}-2y_{8},\\ \tau_{a}{\dot{v}}_{0}=y_{5},\\ \tau_{a}\dot{y_{5}}=A\;S({A\tau_{a}p+C_{2}\tau_{a}v_{1}-C}_{4}\tau_{b}v_{2}-C_{7}\tau_{g}v_{3}+k_{P}I_{ext})-v_{0}-2y_{5},\\ \tau_{a}{\dot{v}}_{1}=y_{6},\\ \tau_{a}\dot{y_{6}}=A\;S(C_{1}\tau_{a}v_{0}+k_{P}I_{ext})-v_{1}-2y_{6},\\ \tau_{b}{\dot{v}}_{2}=y_{7},\\ \tau_{b}\dot{y_{7}}=B\;S(C_{3}\tau_{a}v_{0}+k_{SOM}I_{ext})-v_{2}-2y_{7}. \end{array}$$ (10)

where *k*_*i*_ with *i* = {*P*,*SOM*,*PV*} is the coupling coefficient between the stimulation and the considered subpopulation, governing the impact of *I*_*ext*_(*t*) on the subpopulation. Same generalization holds for the slow and superslow reduced problems given by (7) and (9), respectively.

### Clinical data

Clinical data used for the purpose of this study consisted in SEEG signals collected in a patient with drug-resistant focal epilepsy that required invasive EEG exploration. Recordings were performed using intracranial multichannel electrodes (DIXI Medical, 5–18 contacts; length, 2 mm, diameter, 0.8 mm; 1.5 mm apart), 10 of which are used for investigations. The patient-specific position of each electrode results from a clinical decision process which is based on the many assumptions made from non-invasive data (video EEG recording, semiology, neuro-imaging data, clinical examination). Electrodes were implanted according to the stereotactic method of Talairach [39]. SEEG signals were recorded on a Deltamed system on a maximum number of channels equal to 128, and were sampled at 256 Hz and recorded to hard disk (16 bits/sample) using no digital filter. The only filter present in the acquisition procedure was a hardware analog high-pass filter (cut-off frequency of 0.16 Hz) used to remove very slow variations that sometimes contaminate the baseline. In the patient for which data is displayed in the remainder of the manuscript, a surgical operation was performed 6 month after pre-surgical exploration (cortectomy of the frontal dorsolateral region). The chosen monopolar signal was recorded on one electrode located in the depth of a sulcus located in the dorsolateral frontal cortex. It was chosen because the recorded structure was highly epileptogenic considering the exhibited epileptiform activity (during interictal periods and at the onset of seizures). Histological data revealed the presence of a focal cortical dysplasia (Taylor). After surgery, the patient was seizure free (Engel IA). As a reminder, SEEG is always carried out as part of normal clinical care of patients who give informed consent in the usual way. Patients were informed that their data may be used for research purposes.

### Computational methods

The bifurcation analysis was in done with AUTO-07p [40]. Model equations were implemented in XPPaut [41]. Stochastic differential equations were iterated using Euler-Maruyama method with a step size *dt* = 10^−4^ second. All simulation files are available from the GitHub database (https://github.com/elifkoksal/NMM_BurstingDynamics).

## Results

### Pre-ictal spiking during interictal to ictal transition

**Fig 1:**
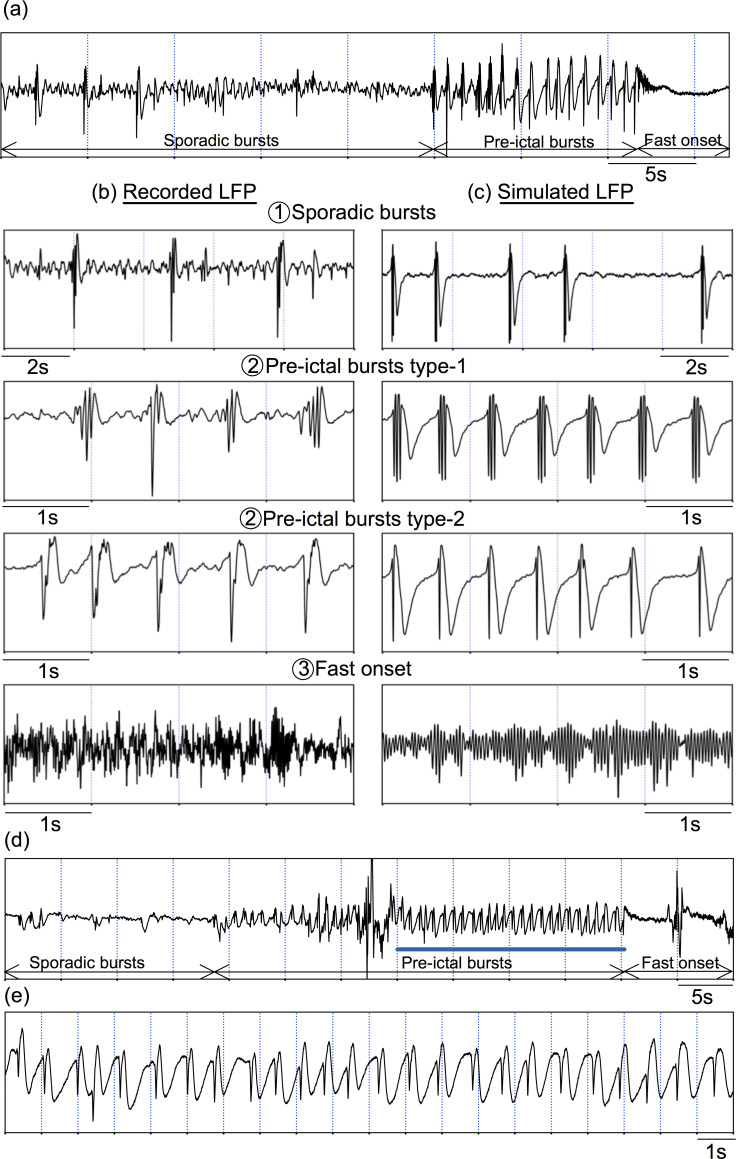
SEEG signals recorded in two patients with epilepsy during the interictal to ictal transition and simulated signals. (a) Epileptic seizure recorded in a patient showing the typical pre-ictal spiking pattern with three phases: sporadic spikes, pre-ictal bursts, and fast onset. (b) Zoom into each phase of the actual SEEG signal. The pre-ictal burst type-1 is followed by the pre-ictal burst type-2. (c) Simulated signals corresponding to each phase. (d) Epileptic seizure recorded in a different patient showing the typical pre-ictal spiking pattern with three phases. The region of pre-ictal bursting is zoomed in panel (e).

In partial (i.e. focal) epilepsies, the onset of seizures is characterized by the appearance of a rapid discharge, typically in the gamma frequency band ([25, 120] Hz) [27]. This fast onset activity has long been recognized as a hallmark of the epileptogenic zone, and a number of methods have been proposed to make use of this pattern to identify the epileptogenic zone [42,43]. Interestingly, fast onset activity can be preceded by a specific electrophysiological pattern consisting of sustained large amplitude bursts with superimposed faster spikes, which can be observed in various etiologies [44]. Two examples of this pre-ictal pattern, as recorded in two different patients during pre-surgical investigation with depth electrodes, are shown in **Fig 1**. As depicted, this dynamical regime starts with sporadic bursts, which become periodic to change into a sustained discharge of pre-ictal bursts. In the first recording in **Fig 1**A, the number of spikes of the bursts gradually decreases during the pre-ictal burst phase, which continues approximately for 14 seconds. In the second example in **Fig 1**D, pre-ictal bursting continues for approximately 35 seconds. In this example, the amplitude of pre-ictal bursts increases. The pre-ictal burst phases are followed by the fast activity that actually marks the onset of the seizure. Patterns of SEEG signals recorded during interictal–ictal transitions for different seizures of a given patient are typical and reproducible [45,46]. As for the patients whose pre-ictal activity are exemplified in **Fig 1**, such a transition is persistent with quantitative variations in 1) duration of the pre-ictal bursting period, 2) amplitude of discharges, and 3) number of fast spikes in the bursts.

System (1) represents a physiologically relevant system that includes neuronal subpopulations of excitatory glutamatergic pyramidal neurons, inhibitory SOM+ and PV+ GABAergic interneurons, and makes average PSPs at the level of each subpopulation accessible. This NMM has been extensively explored to establish relationships between model parameters and electrophysiological patterns observed in SEEG recordings [22,47]. For instance, increasing the ratio of the synaptic gain of the excitatory pyramidal cell population and inhibitory SOM+ interneuron population introduces a region in the parameter space where the system can undergo different stages of epileptogenic activity as a function of the synaptic gain of inhibitory SOM+ interneuron population, parameter *B*, and the synaptic gain of inhibitory PV+ interneuron population, parameter *G*.

**Fig 2:**
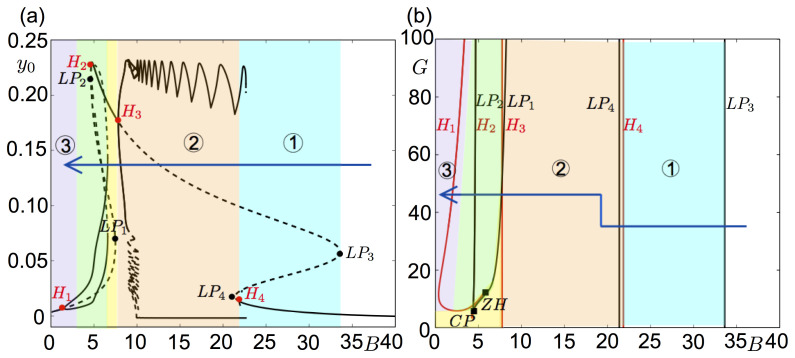
Bifurcation diagrams of the system (1) showing the different pre-ictal stages numbered 1–3 in Fig 1. (a) Amplitude of the PSP of the pyramidal cell subpopulation is plotted as a function of the synaptic gain of the SOM+ interneuron subpopulation *B*. The other system parameters are given in **Table** 1 with *G* = 35. Bold and dashed lines correspond to stable and unstable solutions, respectively. Region 1 (blue) corresponds to sporadic bursts, region 2 (orange) to sustained bursts, and region 3 (purple) to low voltage fast onset activity. The system yields large amplitude ≈30 Hz oscillations in the unnumbered green shaded region. The unnumbered yellow shaded area corresponds to high amplitude stable equilibrium points, and white corresponds to physiological background activity. The arrow shows the route from background to low voltage fast onset activity in the parameter space. In order to preserve the readability of the diagram, the bifurcations along the branches of periodic solutions are not shown. (b) Co-dimension 2 diagram of the Hopf (H) and limit point (LP) bifurcations marked on panel (a) in the parameter space of *B* and *G* (synaptic gain of the PV+ interneuron subpopulation). The LP_1_ and LP_2_ points merge on a cusp (CP) bifurcation, and the H_1_ and H_2_ merge on a zero-Hopf (ZH) bifurcation.

The thorough exploration of system (1) led to the identification of three key parameters: *B*,*G*, and the strength of the excitatory synaptic coefficient on the PV+ interneuron subpopulation *C*_5_. Indeed, the tuning of these three parameters enables replicating of the different pre-ictal stages shown in **Fig 1**A. These results are illustrated by the bifurcation diagram in **Fig 2**A for the parameter set given in **Table** 1. As depicted, the decrease of parameter *B* yields a transition from background activity to fast onset activity, though pre-ictal spiking. **Fig 2**B shows where these activity regions are localized in the (*B*,*G*)-parameter space.

Let us walk through the bifurcation diagram in **Fig 2**A, starting the equilibrium point at *B* = 0, and increase *B*. The system first undergoes a supercritical Hopf bifurcation (H_1_) at *B*≈1.51, giving stable limit cycles at ≈30 Hz (gamma activity). The amplitude of these solutions increases with *B* and they become unstable *via* a saddle node bifurcation of periodic orbits at *B*≈6.56. The branch of periodic orbits connects to a Hopf bifurcation (H_2_) of subcritical type at *B*≈4.56, while the range of oscillations extends until *B*≈6.56. The branch of equilibrium points undergoes two limit point (LP) bifurcations (LP_1_ for *B*≈7.43 and LP_2_ for *B*≈4.55) between H_1_ and H_2_. Then, we identify a third Hopf bifurcation (H_3_) of supercritical type for *B*≈7.74, where a branch of stable oscillations around ≈6 Hz appears. As *B* increases, this branch connects to stable bursting orbits by passing through several limit folds around *B*≈8.86. At *B*≈10 the system reaches to the maximum number of spikes per burst orbit (11 spikes for this parameter set). Increasing *B* decreases the number of spikes *via* the horizontal up-and-downs in *y*_0_ between *B*∈(9.5,22.5). The bursts terminate at *B*≈22.5. The branch holding the unstable equilibrium points forms a Z-shaped curve with two folds at *B*≈21.3 (LP_3_) and *B*≈35.6 (LP_4_), with unstable focus on the upper branch, saddles in the middle and stable nodes on the lower branch after a subcritical Hopf bifurcation (H_4_) which gives unstable limit cycles making a heteroclinic connection with the middle branch. For *B*>35.6, the system only has stable equilibrium points as solutions in the unnumbered white area, which corresponds to physiological background activity.

Continuation of the LP and Hopf bifurcations marked on **Fig 2**A in the (*B*,*G*)-space is shown in **Fig 2**B. It can be seen that the locations of LP_3_, LP_4_, H_3_, H_4_ points do not depend on *G*, whereas the locations of LP_1_, LP_2_, H_1_ and H_2,_ which are related to the oscillations at ≈30 Hz, do. The fast onset region does not exist for small values of B if *G*<5, for which the system only has equilibrium points for *B*<*B*_*H*3_. As *G* increases, the system undergoes Hopf bifurcations (H_1_ and H_2_) that introduce gamma-band fast oscillations (for G>5). The amplitude of these fast oscillations are smaller closer to H_1,_ and this difference is more visible in simulated LFPs. Under stochastic inputs, the system can yield fast oscillations for *B*∈(0, *B*_*H*1_), and a mixture of fast and slow oscillations of ≈6 Hz for *B*∈(*B*_*H*2_, *B*_*H*3_). Furthermore, *G* controls the amplitude of the spikes of bursting solutions, which increases with *G*.

Assume that system (1) is initially in the background activity mode, which corresponds to the white region in **Fig 2**A for *B*>35.6, where unique stable equilibrium points on the bifurcation curve is observed. In the blue region between the two folds LP_3_ and LP_4_, the bifurcation curve takes a Z-form with stable nodes on the lower branch, unstable nodes in the middle and saddle-nodes on the upper branch. For *B* values in this blue region, system (1) under a stochastic input *p*(*t*) undergoes sporadic bursts, with an increasing probability as *B* approaches to the left fold. As *B* decreases, the system enters into the bursting region (orange region). Note that further increasing B would increase the number of spikes. However, in the recordings we see that the number of spikes decreases in the course of the pre-ictal bursting regime as the system approached the low voltage fast onset (LVFO), transition from type-1 to type-2 bursting. This change is very subtle to be reproduced in the model because, as detailed in the Sec. *Burst analysis*, the number of spikes depends on the presynaptic potential on PV+ interneurons: the lower it is, the more spikes within the burst are obtained. Thus, the number of spikes increases when the inhibitory input decreases, or when the excitation onto PV+ interneurons increases. At this stage, transition from the type-1 bursting to type-2 bursting is obtained by keeping *B* constant, but decreasing the excitatory post-synaptic potential (EPSP) on PV+ by decreasing progressively *C*_5_ to 300 to reduce the number of spikes; and increase *G* to increase spikes amplitude. Under these variations, the bifurcation diagram in **Fig 2**A remains qualitatively the same, the most important quantitative change being the location of the Hopf bifurcation point H_1_ related to the LVFO. As shown in **Fig 2**B, increasing *G* moves the H_1_ towards right along the *B*-axis, and initiates the LVFO for higher values of *B*≈*B*_*H*1_. Under stochastic input, one can observe LVFO for *B*≤*B*_*H*1_, as well (purple regions in **Fig 2**). The large amplitude fast oscillations that are obtained for higher *B* values in (*B*_*H*1_, *B*_*H*2_) can also be observed in SEEG recordings during the transition to epileptic seizures.

### Bursting analysis

**Fig 3:**
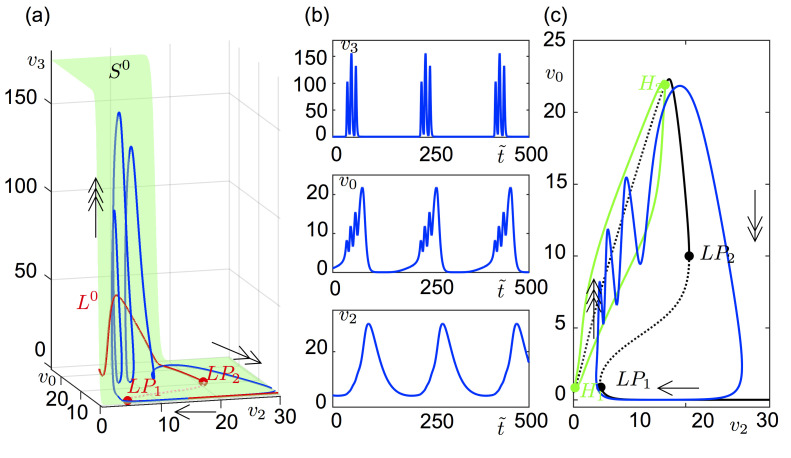
Bursting orbit of system (3). (a) Solution of (3) (blue orbit) and *L*^0^ (red curve) on the critical surface *S*^0^(green surface) projected on the (*v*_0_, *v*_2_, *v*_3_)-space. Single-headed, double-headed and triple-headed arrows indicate the flow direction during superslow, slow and fast time-scales, respectively. LP denotes limit point bifurcation. The *L*^0^ curve changes stability at two limit points, *LP*_1_ and *LP*_2_ (red dots). The middle branch of the *L*^0^ curve between these limit points is unstable (dashed). (b) Time course of the variables (*v*_3_, *v*_0_, *v*_2_) of the orbit plotted in panel (a). (c) Solution of (3) projected on the bifurcation diagram (black curve) of (4) for ε = 0 where *v*_2_ is threaded as a parameter. Arrows show the direction of the flow with respective time-scales. Bold and dashed lines correspond to stable and unstable solutions, respectively. H donates a Hopf bifurcation, LP a limit point bifurcation. The equilibrium points along the black Z-shaped curve are unstable on the middle branch of the curve, between *LP*_1_ at $v_{2}^{LP_{1}}=4.778$ and *LP*_2_ at $v_{2}^{LP_{2}}=20.66$ (black dots), and on the upper branch between H_1_ at $v_{2}^{H_{1}}=0.27$ and H_2_ at $v_{2}^{H_{2}}=14.27$ (green dots). The amplitude of the stable limit cycles is bounded by the green continuous curves connecting the *H*_1_ and *H*_2_ in the ε = 0 limit.

We investigate the bursting dynamics of system (1) using system (3), which is a kind of nondimensionalized version of (1) but expressed in an explicit slow-fast formulation. **Fig 3**A shows a bursting solution of (3) in the (*v*_0_, *v*_2_, *v*_3_)-space, the critical manifold *S*^0^ and the superslow manifold *L*^0^ (see Sec. *Slow-fast formulation of the model* for definition). The critical manifold *S*^0^ is normally hyperbolic (not folded) and stable, and stretches between almost horizontal surfaces (lower and upper) with an almost vertical plane. The superslow manifold *L*^0^ has branches both on the lower horizontal surface and vertical surface of *S*^0^. While the part of *L*^0^ on the vertical surface of *S*^0^ is stable, the part on the lower horizontal surface of *S*^0^ is divided into stable and unstable sections at two fold points LP_1_ and LP_2_. The curve *L*^0^ is stable along the branch that is almost parallel to the *v*_2_-axis, unstable along the branch between LP_1_ and LP_2_, and then becomes stable again. The stable and unstable branches of *L*^0^ are normally hyperbolic, whereas the fold points LP_1_ and LP_2_ are not.

The critical manifold *S*^0^ and superslow manifold *L*^0^ perturb for small enough values of time-scale parameters, hence the three-time-scale dynamics of (3) approximate to *S*^0^ and *L*^0^. During the superslow time-scale under (9), the bursting orbit follows the stable branch of *L*^0^ almost parallel to the *v*_2_-axis. Near the fold point LP_1_, the trajectory bends in the *v*_3_-direction along the vertical plane of *S*^0^ and enters into the spiking regime, which runs in fast time-scale. The spiking terminates close to LP_2_ and the trajectory jumps back to the stable branch of *L*^0^ almost parallel to the *v*_2_-axis in slow time-scale under (8). **Fig 3**B shows the time series in $\tilde{t}$ of the orbit in **Fig 3**A.

**Fig 4:**
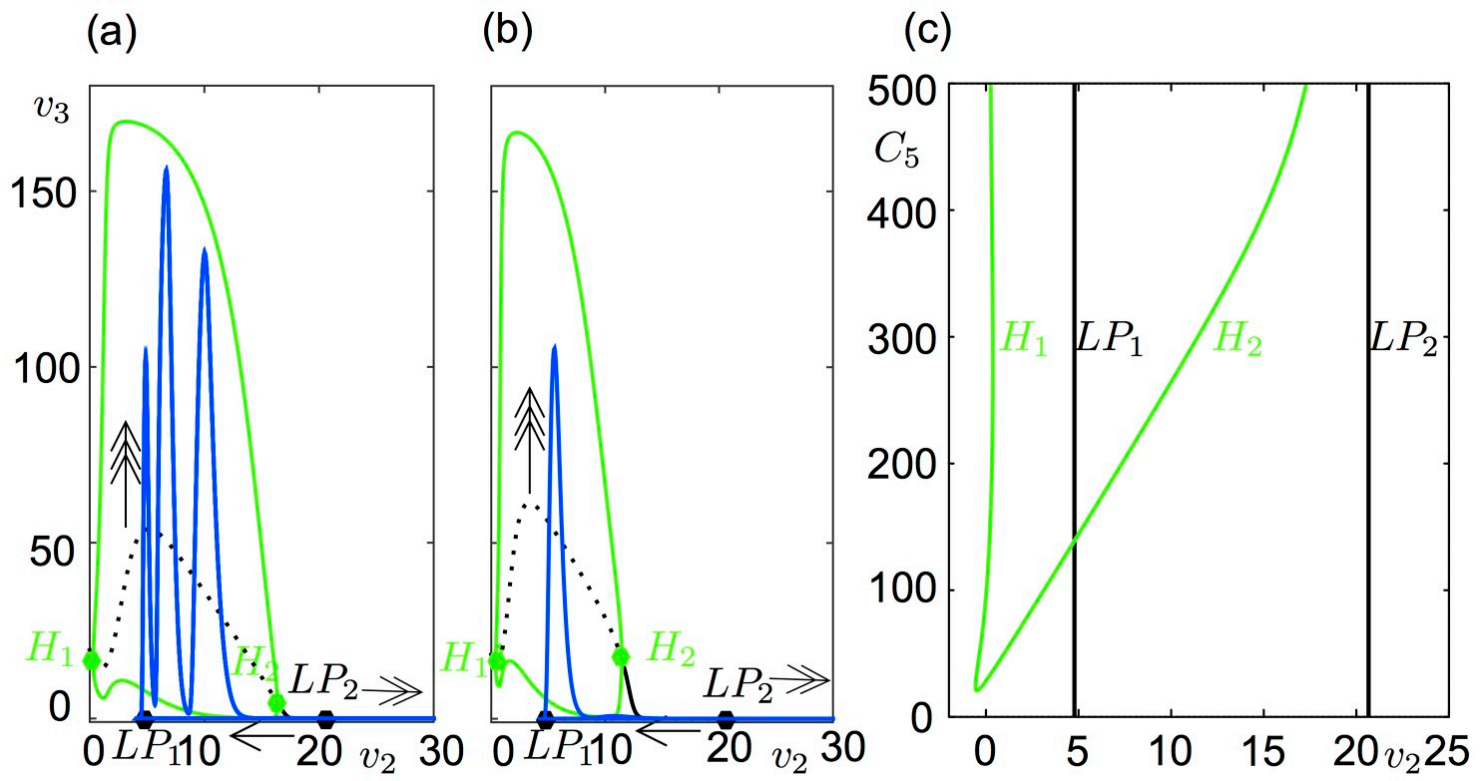
Spike number as a function of *C*_5_. (a) Solution of (4) with 3 spikes for *C*_5_ = 500 projected on the bifurcation diagram of the fast system (5) as a function of *v*_2_. Arrows indicate the direction of the flow. (b) Solution of (4) with 1 spike for *C*_5_ = 300 projected on the bifurcation diagram of the fast system (5) as a function of *v*_2_. Arrows indicate the direction of the flow. (c) Co-dimension 2 diagrams of the Hopf (H) points (green) and the limit points (LP, black) in the (*v*_2_, *C*_5_) parameter space marked on the left and middle panels. As *C*_5_ decreases, H_2_ moves leftwards and eventually the spike number decreases. For *C*_5_ = 139, H_2_ and LP_1_ are aligned at *v*_2_ = 4.778. A further decrease in *C*_5_ places H_2_ on the left of LP_1_ and leaves no chances for a bursting solution.

For a better understanding of the bursting dynamics, we consider system (4) at *ε* = 0 for which the variables of the slowest subsystem (*v*_2_, *y*_7_) act as parameters of the (*v*_3_, *y*_8_, *v*_0_, *y*_5_, *v*_1_, *y*_6_)-subsystem. Since only *v*_2_ appears in the (*v*_3_, *y*_8_, *v*_0_, *y*_5_, *v*_1_, *y*_6_)-subsystem, its dynamics depend on *v*_2_. In **Fig 3**C, the bursting orbit in **Fig 3**A is superimposed on the bifurcation diagram of the (*v*_3_, *y*_8_, *v*_0_, *y*_5_, *v*_1_, *y*_6_)-subsystem in (4) at *ε* = 0 as a function of *v*_2_. Although the fastest variables of (4) are (*v*_3_, *y*_8_), we chose *v*_0_ vs *v*_2_ for a clearer visualization (the same trajectory and the bifurcation diagram are given on the (*v*_3_, *v*_2_)-plane **Fig 4**). We see that the corresponding system poses a Z-shaped bifurcation diagram as a function of *v*_2_ with two folds, $v_{2}^{LP_{1}}$ and $v_{2}^{LP_{2}}$. The equilibrium points are stable on the lower branch of the Z-shaped curve for $v_{2}>v_{2}^{LP_{1}},$ unstable along the middle branch between $v_{2}^{LP_{1}}$ and $v_{2}^{LP_{2}}$. The upper branch has two supercritical Hopf bifurcations, at $v_{2}^{H_{1}}$ and $v_{2}^{H_{2}}$, with stable limit cycles in between. Along the upper branch, equilibrium points are stable for $v_{2}<v_{2}^{H_{1}}$ and $v_{2}^{H_{2}}<v_{2}<v_{2}^{LP_{2}}$. The bursting behavior resulting from this bifurcation structure in the (*v*_3_, *y*_8_, *v*_0_, *y*_5_, *v*_1_, *y*_6_)-subsystem is classified as ‘fold/Hopf bursting’ by Izhikevich [32] due to the presence of a ‘fold/Hopf’ hysteresis in the bifurcation diagram.

System (4) may undergo through these bifurcations in a repetitive manner for *ε*≠0, which results eventually in the bursting solutions for small enough values of *ε*. As the arrows on **Fig 3**C and the traces on **Fig 3**B demonstrate, the trajectory follows the lower stable branch during the quiescence phase of the bursting, which terminates near $v_{2}\approx v_{2}^{LP_{1}}$. Then, it jumps to the region of the stable limit cycles on the upper branch, which initiates the active phase of the bursting. The spiking frequency during the active phase is faster at the beginning than the end due to the fact that the Hopf bifurcation at $v_{2}^{H_{1}}$ gives limit cycles with ≈30 Hz frequency whereas the Hopf bifurcation at $v_{2}^{H_{2}}$ gives limit cycles with ≈10 Hz. The spiking terminates at $v_{2}\approx v_{2}^{H_{2}}$, but the active phase continues until the trajectory jumps back to the stable lower branch at $v_{2}\approx v_{2}^{LP_{2}}$. We underline that as *ε*→0, the bursting orbit attaches more and more the bifurcation diagram obtained for *ε* = 0 (see **S2 Fig** for an example).

The main difference between the type-1 and type-2 bursting is the number of spikes during the active phase of bursting. In the model, the variations in the number of spikes can be met by changing the excitation level on the PV+ interneurons: as aforementioned, the number of spikes increases with the amount of excitation received by PV+ interneurons. This can be achieved by either decreasing inhibition or by increasing excitation. For instance, decreasing *B* in region-2 in **Fig 2** increases the number of spikes. In (4) at *ε* = 0, the excitation on PV+ depends on two synaptic coupling coefficients, *C*_5_ and *C*_6_. The effect of *C*_6_ will be similar to the one of *B*, since they both scale the PSP of SOM+ interneurons given by the variable *v*_2_ in (4). Below, the role of the excitatory synapses in the (*v*_3_, *y*_8_, *v*_0_, *y*_5_, *v*_1_, *y*_6_)-subsystem by changing *C*_5_ is investigated.

As displayed by **Figs** **3**C, **4**A and **4**B, the spikes are bounded by LP_1_ and H_2_ in the bifurcation diagram of the (*v*_3_, *y*_8_, *v*_0_, *y*_5_, *v*_1_, *y*_6_)-subsystem as a function of *v*_2_. The distance between LP_1_ and H_2_ in *v*_2_ affects the number of spikes; the further they are, the more spikes the burst has. In **Fig 4**C, LP and Hopf bifurcations are continued in the parameter space of (*v*_2_, *C*_5_). While the LP_1_ and LP_2_ lie along almost vertical lines, the Hopf bifurcation points form a V-shaped curve along which the left arm locates the H_1_ points and the right arm the H_2_ points. The distance between H_2_ and LP_1_ increases with *C*_5_, hence, the spike number. At *C*_5_ = 139, H_2_ and LP_1_ are aligned. Further decrease in *C*_5_ places H_2_ on the left of LP_1_ and leaves no chances for a bursting solution. The system yields only relaxation type of oscillations for *C*_5_<139.

Overall, the aforementioned analysis shows that pre-ictal bursting runs in three-time-scales. The system sustains the bursting regime for a certain range of parameter *B* denoting SOM+ synaptic gain. The complex pre-ictal bursting pattern can be accurately adjusted by tuning parameters *G*, which controls the PV+ synaptic gain, and the connectivity coefficient *C*_5_, which controls PV+ excitability. In particular, the number of spikes and their amplitude can be adjusted by tuning *C*_5_ and *G*, respectively.

### Strong perturbation analysis

The mean membrane potential of a neuronal subpopulation can be altered by electrical and optical stimulations. Under the assumption of an additive model for the stimulation effect, an external input can be included in the ‘pulse-to-wave’ functions, *S*(*v*), of the NMM after being scaled by the subpopulation specific impact coefficients (see Sec. *Strongly perturbed system*).

A pulse input (biphasic or monophasic) changes the PSP of the perturbed subpopulation by shifting it above its base level *S*(0). We assume that a neural mass block, given by $\ddot{y}=W/\tau_{w}S(I_{ext}(t))-2/\tau_{w}\dot{y}-1/\tau_{w}^{2}y$, receives biphasic stimulation. The PSP of the neural mass block increases during the anodal pulse (positive perturbation or depolarization of the membrane potential), but decreases (discharges) during the cathodic pulse (negative perturbation or hyperpolarization of the membrane potential) and between the inter-pulse intervals of the biphasic input. Depending on the pulse width, pulse amplitude, and mostly on the synaptic time constant of the neural mass block, this shift may be sustained or not. For instance, the discharge takes longer in a neural mass block with slow synaptic kinetics than the one with fast synaptic kinetics. If the pulse frequency is sufficiently high to stimulate the neural mass before it completely resumes to its base level, then the PSP of the neural mass can oscillate above the base level. As visualized in **S3 Fig**, the same perturbation shifts the PSP of a neural mass with slow synaptic kinetics, while the neural mass with fast synaptic kinetics decays to its base level during the inter-pulse intervals of the stimulation. Increasing the stimulation frequency can keep the PSP of the neural mass with fast kinetics above the base level, and therefore the firing rate and PSP of the fast neural mass increase with the stimulation frequency.

The bursting solution is driven by the slow oscillations in system (3) (see Sec. *Bursting Analysis*). The slow dynamics of (3) (subsystems representing the pyramidal cell and SOM+ interneuron subpopulations) can be approximated by (7), which preserves the burst-driver slow oscillations behavior for the same parameter values yielding bursting oscillations in (3) (see Sec. *Slow-fast formulation* and **S2 Fig**). Thus, it is sufficient to investigate the response of (7) under perturbation to understand the impact of the perturbation on the burst-driver slow dynamics. The most common signal delivered to brain tissue in the field of deep brain stimulation (DBS) is bi-phasic pulses with balanced anodic/cathodic phases of brief durations (approximately 100 μs). Below, the impact of anodic and cathodic constant external inputs is considered without taking into account their duration, to simply understand how they alternate the phase space of system (7). For this purpose, a constant input (*I*_*ext*_ = 1) scaled with the impact coefficients *k*_*P*_ and *k*_*SOM*_ is applied to (7) by following the formulism given by (10) (see Sec. *Strongly perturbed system*).

**Fig 5:**
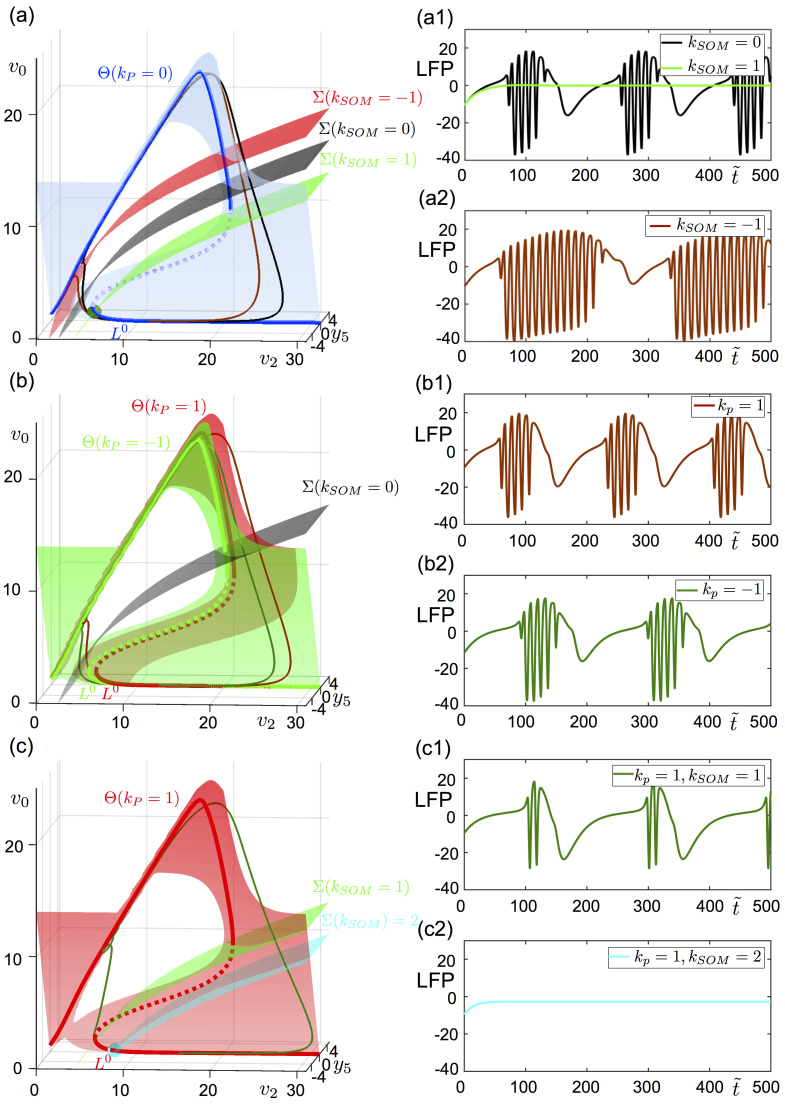
Geometrical analyses of a constant input. Constant input is applied to SOM+ interneurons (a), to pyramidal cells (b) and to both SOM+ interneurons and pyramidal cells (c). Left panels show the projection of the nullsurfaces, critical slow manifold and the orbit of the reduced model (7). Right panels show the LFP signal of the full system (3) subject to the constant inputs analyzed on the left. All parameters are as given in Table 1, except *B* = 15. (a) The *y*_5_-nullsurface Θ (blue surface for *k*_*P*_ = 0), and *y*_7_-nullsurface Σ (red surface for *k*_*SOM*_ = −1, black surface for *k*_*SOM*_ = 0, green surface for *k*_*SOM*_ = 1) are projected on the (*v*_2_, *y*_5_, *v*_0_)-space. The blue curve *L*^0^ (stable on the bold, unstable on the dashed) is on the intersection between Θ and the {*y*_5_ = 0}-hyperplane. The black and red orbits are the solutions of the system for *k*_*SOM*_ = 0 and *k*_*SOM*_ = −1, respectively. For *k*_*SOM*_ = 1, the solution approaches to the green stable equilibrium point on the intersection between Σ(*k*_*SOM*_ = 1) and *L*^0^. Panel (a1) shows the time series for *k*_*SOM*_ = {0, 1}, and panel (a2) for *k*_*SOM*_ = −1. (b) The *y*_5_-nullsurface Θ (red surface for *k*_*P*_ = 1, green surface for *k*_*P*_ = −1), and *y*_7_-nullsurface Σ (black surface for *k*_*SOM*_ = 0) are projected on the (*v*_2_, *y*_5_, *v*_0_)-space. The red curve *L*^0^ (stable on the bold, unstable on the dashed) is on the intersection between Θ(k_P_ = 1) and the {*y*_5_ = 0}-hyperplane. The green curve *L*^0^ (stable on the bold, unstable on the dashed) is on the intersection between Θ(k_P_ = −1) and the *y*_5_ = 0 hyperplane. The green and red orbits are the solutions of the system for *k*_*P*_ = 1 and *k*_*P*_ = −1, respectively. Panel (b1) shows time series for *k*_*P*_ = 1, and panel (b2) for *k*_*P*_ = −1. (c) The *y*_5_-nullsurface Θ (red surface for *k*_*P*_ = 1) and *y*_7_-nullsurface Σ (green surface for *k*_*SOM*_ = 1, blue surface for *k*_*SOM*_ = 2) are projected on the (*v*_2_, *y*_5_, *v*_0_)-space. The red curve *L*^0^ (stable on the bold, unstable on the dashed) is on the intersection between Θ(k_P_ = 1) and the *y*_5_ = 0 hyperplane. The green curve *L*^0^ (stable on the bold, unstable on the dashed) is on the intersection between Θ(k_P_ = 1) and the {*y*_5_ = 0}-hyperplane. The green orbit is the solution of the system for (*k*_*P*_, *k*_*SOM*_) = (1,1). For (*k*_*P*_, *k*_*SOM*_) = (1,2) the solution approaches to the cyan stable equilibrium point on the intersection between Σ(*k*_*SOM*_ = 2) and *L*^0^. Panel (c1) shows time series for (*k*_*P*_, *k*_*SOM*_) = (1,1), and panel (c2) for (*k*_*P*_, *k*_*SOM*_) = (1,2).

In **Fig 5**, subpopulations representing pyramidal cells and inhibitory SOM+ interneurons are perturbed. The left panels of **Fig 5** show the *y*_5_-nullsurface Θ, *v*_2_-nullsurface Σ and the superslow manifold *L*^0^ projected on the (*y*_5_, *v*_2_, *v*_0_)-space. The solution of (7) is visible on the left panels, and the solution of (3) for the same parameters is given on the right panels of **Fig 5**. **Fig 5**A shows the case where only the SOM+ interneuron subpopulation described by the (*v*_2_, *y*_7_)-subsystem in (7) is subject to the constant external input (*k*_*P*_ = 0). In the absence of any perturbation (*k*_*SOM*_ = 0), Θ and Σ intersect for *v*_2_>20. System (7) has a limit cycle which flows on Θ and (3) a burst orbit (black solutions **Fig 5**A and **5**A1, respectively). The quiescence phase of the burst corresponds to the slow passage following *L*^0^ where *v*_0_≈0, and the active phase correspond to the trajectory on the upper sheet of Θ.

A key point in terms of controlling bursting activity through direct stimulation is that an input leading to a bifurcation from the stable limit cycle to an equilibrium point can prevent the system from bursting by keeping the system in the silent phase. This can be achieved by an input that ensures an intersection between Σ hyperplane and the lower branch of *L*^0^. Indeed, for *k*_*SOM*_ = 1, (7) possesses a stable equilibrium point near the left fold of *L*^0^ which traps the trajectory (green dot in **Fig 5**A). For the same input, the bursting in (3) is aborted (green solution in **Fig 5**A1). On the other hand, a negative constant input (*k*_*SOM*_ = −1), moves Σ away from the left fold of *L*^0^. Being Σ closer to the upper branch of *L*^0^ prolongs the active phase of the burst and increases the number of spikes, as seen in **Fig 5**A2. These observations indicate that increasing the excitation on SOM+ interneurons can abort bursting.

In **Fig 5**B, only the subsystem representing the pyramidal cells receives the perturbation (*k*_*SOM*_ = 0). The input on the pyramidal cell subpopulation acts on Θ. While positive constant input (*k*_*P*_ = 1) increases the distance between the lower fold of *L*^0^ and Σ, negative constant input (*k*_*P*_ = −1) decreases this distance. Both systems (7) and (3) preserve the oscillatory behavior for these values of *k*_*P*_, yet, the oscillation frequency decreases for *k*_*P*_ = −1 due to the decreased distance between the lower fold of *L*^0^ and Σ). Thus, hyperpolarization of pyramidal cells by increasing inhibition on them can abort bursting.

As aforementioned above, pulsed stimulation increases the firing rate of a neuronal population. However, a stimulation applied to one specific region might not affect all neural populations in the same manner. This can be due to the relative position of electrodes with respect to neurons, cell specific firing thresholds, or synchronization level within neural subpopulations. However, such features can bring certain advantages in aborting bursting. **Fig 5**C shows the response of the system when both subpopulations of pyramidal cells and SOM+ interneurons are perturbed, the oscillatory behavior in systems (7) and (3) continues under the same positive constant input (*k*_*P*_ = *k*_*SOM*_ = 1). With such input, the number of spikes during the active phase is decreased (**Fig 5**C1). If the subpopulation of SOM+ interneurons is perturbed more strongly than the subpopulation of pyramidal cells (*k*_*P*_ = 1, *k*_*SOM*_ = 2), the system can bifurcate to the resting state (**Fig 5**C2).

Although the reduced system (7) does not include the fast dynamics of the PV+ interneurons, the effect of the perturbation on the subpopulation of PV+ interneurons can be understood geometrically. First, let us notice that increasing the inhibition on SOM+ interneurons encourages spiking (**Fig 5**A and **5**A2), while increasing the excitation on SOM+ aborts bursting (**Fig 5**A and **5**A1). Perturbing (stimulating) the subpopulation of PV+ interneurons increases the PSP from PV+ interneurons to pyramidal cells, reduces the PSP from pyramidal onto SOM+, and in turn favors bursting. Another way to illustrate the impact of perturbing the PV+ on bursting is to examine the diagram in **Fig 4**. Anodic pulses can shorten the quiescent phase in the ‘fold/Hopf’ hysteresis loop by kicking the trajectory to the region of stable limit cycles between the two Hopf bifurcations H_1_ and H_2_. Hence, such pulses applied periodically can increase the bursting frequency by shortening the quiescent phase. On the other hand, cathodic pulses can lengthen the quiescent phase by hooking the trajectory near the down state of the hysteresis loop. One can also think of inhibitory effect of PV+ subpopulation on the pyramidal cell population which could abort bursting. Indeed, it would be possible if an input was capable of depolarizing the PV+ subpopulation during the quiescence phase of the bursting where it is inhibited by the SOM+ subpopulation. To overcome the inhibition from the SOM+ subpopulation to the PV+ subpopulation, such an input should be very high in amplitude (order of 20 if a constant input on the PV+ subpopulation is considered), which could induce undesired discharges.

Overall, this geometric perturbation analysis helps to clarify the role of hyperpolarizing and depolarizing inputs on ongoing bursting activity. In particular, depolarization of the subpopulation of SOM+ interneurons or hyperpolarization of the subpopulation of pyramidal cells can abort bursting by keeping the sum of PSPs at low levels. Depolarization of the subpopulation of PV+ interneurons contributes to bursting.

### Stimulation applied during the pre-ictal burst regime

The analysis in Sec. *Perturbation Analyses* has shown that a positive constant input applied to the subpopulation of SOM+ interneurons can bifurcate the limit cycle (oscillating epilepsy-like activity) to an equilibrium point (background activity), while a positive constant input on the subpopulations of pyramidal cells and PV+ interneurons preserve bursting and high frequency oscillations (**Fig 5**). Hence, an appropriate strategy for pre-ictal bursting abortion consists in the excitation of the SOM+ interneuron subpopulation.

**Fig 6:**
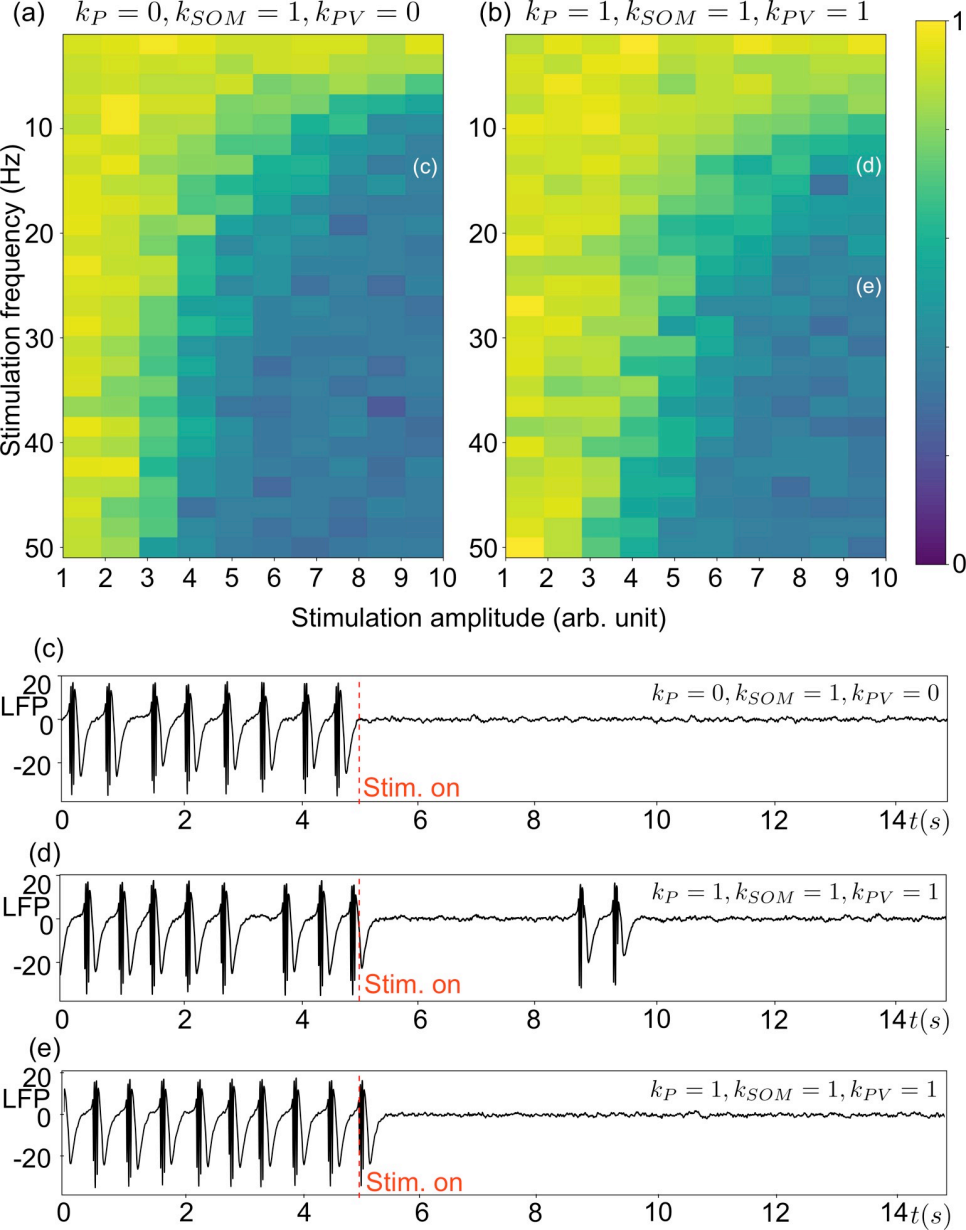
System (1) under stimulation. Biphasic stimulation with a 0.5 ms pulse width (total pulse duration is 1 ms) is applied to the system in type-1 bursting. Panels (a) and (b) show the energy map of the simulated LFP signal that is lower in the blue region than the yellow region (see the color bar on the right). The energy of the LFP signal was computed by using $E=\sum_{n=1}^{n=N}{\left|x(n)\right|}^{2},$ where *n* stands for the index of and *N* for the size of the discrete signal *x*(*n*). (a) Only the SOM+ interneurons receive the biphasic perturbation. (b) The pyramidal cell, SOM+ interneurons and PV+ interneurons receive the same biphasic perturbation. Panels (c), (d) and (e) show the time course of the marked stimulation on panels (a) and (b). (c) 15 Hz biphasic stimulation with 10 arb. unit amplitude is applied to the SOM+ interneurons (*k*_*SOM*_ = 1, *k*_*P*_ = *k*_*PV*_ = 0). (d) 15 Hz biphasic stimulation with 10 arb. unit amplitude is homogenously applied to all subpopulations (*k*_*SOM*_ = *k*_*P*_ = *k*_*PV*_ = 1). (e) 25 Hz biphasic stimulation with 10 arb. unit amplitude is homogenously applied to all subpopulations (*k*_*SOM*_ = *k*_*P*_ = *k*_*PV*_ = 1).

In this section, the results obtained from the mathematical analysis are translated into an *in silico* set-up mimicking experimental conditions. Typically, charge-balanced bi-phasic pulses (pulse width = 0.5 ms and total duration 1 ms) with an arbitrary unit (arb. unit) amplitude are applied during the pre-ictal bursting/spiking regime in the presence of a stochastic input. In order to test our predictions on the role of different neural populations, only SOM+ interneurons are perturbed in **Fig 6**A (*k*_*SOM*_ = 1, *k*_*P*_ = 0, *k*_*PV*_ = 0), whereas in **Fig 6**B all neural subpopulations are perturbed homogenously (coupling coefficients *k*_*SOM*_ = *k*_*P*_ = *k*_*PV*_ = 1).

Results indicate that pre-ictal bursts frequency decreases when the stimulation is switched on at, typically at the instant *t* = 5*s* in both cases. The bursting regime can be aborted if the stimulation frequency and amplitude are sufficiently high. The minimum values of the stimulation frequency and amplitude to abort bursting depend on which neuronal subpopulation receives the stimulation. When only the SOM+ interneuron subpopulation is stimulated, the minimum stimulation frequency and amplitude required to abort bursting are lower than the case where all neural subpopulations are stimulated homogenously. As exemplified in **Fig 6**C, bursting is suppressed at f = 15 Hz for an amplitude of 10 arb. unit when only SOM+ interneurons are stimulated. While the same stimulation can considerably decreases the frequency of bursting events (**Fig 6**D) when all subpopulations are impacted, the stimulation frequency should be increased to 25 Hz for a complete bursting suppression (**Fig 6**E). For a sufficiently high stimulation frequency for which bursting is aborted, increasing the stimulation amplitude can depolarize the PV+ subpopulation. However, such an input induces high frequency components to the burstless response observed in the LFP, as opposed to the low amplitude response which is closer to the physiological background activity (data now shown). While the purpose of the stimulation is not only aborting bursting but also keeping the system as close as possible to background activity, low amplitude stimulations result in more beneficial outputs for clinical applications.

The difference between type-1 and type-2 bursting is the number of spikes during the active phase that is related to the excitatory input onto PV+ interneurons. In particular, the EPSP is larger in the former case. Despite this difference, the bursting mechanisms in both types are the same; i.e. slow oscillations in the SOM+ interneurons drive sequentially and periodically the same type of bifurcations in the subsystem of pyramidal cells and PV+ interneurons. Hence, the strategy for aborting bursting relying on aborting oscillations in the SOM+ subsystem does not depend on the bursting type. The estimations on the stimulation parameters (in terms of frequency and amplitude) given in **Fig 6**C, which are for type-1 bursting, are capable of aborting type-2 bursting and sporadic bursting, as well, because both of the regimes are less excited than type-1 bursting.

## Discussion

Epilepsy is a dynamic and complex disease running on different time-scales [48–50]. Epileptic activity is characterized by long interictal periods (outside seizures), during which the brain behaves mostly as a normal brain, then marked by brief ictal episodes (seizures). The seizure onset, i.e. the transition from interictal to ictal activity, has a wide repertoire in human focal epilepsies [44,51]. In this study, we focused on a specific electrophysiological pattern generally referred to as “pre-ictal spikes” or “pre-ictal discharges”, which has been particularly described in mesial temporal lobe seizures [12–14] but that may also be observed as a seizure onset pattern in neocortical seizures from various origins [44,52]. This complex pattern is signed by large amplitude fast spikes followed by a slow discharge, thus holding the properties of a bursting and is called “pre-ictal bursting” in this paper.

We successfully reproduced the complex pre-ictal bursting pattern in a NMM featuring three subsets of neurons (subpopulations of pyramidal neurons, SOM+ and PV+ interneurons) in [22]. The slow-fast formulation of the model unveiled its three-time-scale structure and the following analysis detailed the mechanisms responsible for the pre-ictal bursting. In particular, the bursting process in the model arose from a high level of excitation among pyramidal neurons as well as onto the PV+ interneuron subpopulation. In the bursting regime, the slow oscillations mediated by the SOM+ interneurons are the drivers of bursting solutions, and the number of spikes during an active phase of a burst depends on the level of excitation on the PV+ interneurons. Ultimately, we showed that a perturbation that was able to stop the slow oscillations in the SOM+ interneuron subpopulation would be sufficient to stop pre-ictal bursting activity.

These model predictions corroborate some experimental findings. Indeed, *in vitro* data from human specimen suggested that a glutamate-dependent cellular and/or synaptic plasticity process underlies the occurrence of pre-ictal discharges during the transition to seizure. Pre-ictal discharges would initiate changes in glutamatergic and GABAergic signaling in groups of neurons larger than those involved in interictal discharges. Repeated discharges would result from a dynamic process that ultimately leads to ictal events [53]. Along the same line, as extensively reviewed in [54], both excitatory and inhibitory networks are involved in epileptogenesis and seizure generation. In particular, GABAergic-mediated mechanisms contribute to synchronizing neuronal networks in epileptic brain structures. Notably, interneuronal activity is enhanced and synchronized during sustained epileptic spikes [55,56].

This viewpoint is particularly interesting if the role of the GABAergic system in the suppression of epileptiform pre-ictal activity is considered when direct brain stimulation applied during the interictal period. For instance, optogenetic stimulation of the CA3 region of hippocampus leads to considerable reduction of seizures in the CA3 region by entrainment of GABAergic interneurons targeting GABA_A_ receptors [57,58]. Low-frequency stimulation of fiber tracts during the inter-ictal period has also been shown to reduce seizures through activation of the GABA_B_ signaling in animal models of temporal lobe epilepsy activity [59–61], as well as with the application of an electrical field [62]. The success of low-frequency stimulation of fiber tracts in focal cortical seizures has also been linked to GABAergic effects [63,64].

Our results are in line with the above reported data, and indicate that an abortive stimulation of the epileptic activity during the pre-ictal bursting regime should primarily target the GABAergic system (mostly on interneurons with slow synaptic kinetics). Stimulating the GABAergic system yielded more pronounced effect as compared with the stimulation pyramidal neurons. The stimulation frequency required to change the PSPs of neural subpopulations was directly linked with their kinetics: the slower they are, the lower stimulation frequency needs to be. At this point, SOM+ interneurons were impacted more than other subpopulations, since SOM+ interneurons have the slowest synaptic kinetics among the considered neuronal types in the model. Besides, the model structure privileges the SOM+ subpopulation to be depolarized by an external stimulation since the SOM+ subpopulation does not receive any IPSP from other subpopulations. Increasing the stimulation frequency would depolarize the SOM+ subpopulation further, thus reinforcing the global inhibition generated by the SOM+ subpopulation. In addition, it has been estimated that a single GABAergic cell may affect more than a thousand pyramidal cells [65,66], which may explain how the activation of GABAergic neurons may become predominant and exert powerful anti-epiletic effects.

Another prediction of this study is the contributing role of PV+ interneurons stimulation on pre-ictal bursting. More specifically, depolarizing the subpopulation of PV+ interneurons contributes to bursting by increasing the number of spikes during the active phase. Also, as it was discussed above, anodal pulses on PV+ interneurons can prompt the active phase and increase the frequency of pre-ictal bursts. Such observation is in agreement with a previous study by Assaf and Schiller [67], in which optogenetic activation of PV+ interneurons in the ictal regime had an anti-epileptic effect, but a pro-epileptic effect when they were activated in the inter-ictal regime. More recently, it was discussed that paradoxical effects of PV+ activation shown in [68] could be related to the timing of the neurostimulation [69]. Therefore, our results support that a precise, on-demand (closed-loop) stimulation system is required to deliver stimulation at an optimal timing, and avoid the promotion of epileptiform activity. The properties of epileptic discharges during the ictal phase are different than the ones of the pre-ictal phase considered in this study. Thus, seizure abortion during the ictal phase may require different stimulation protocols. Investigation of suitable targets and signal waveforms can be considered as a possible avenue for future work.

DBS for epileptic patients is an ongoing research topic, and unfortunately, the lack of randomized control trials comparing different stimulation protocols hampers obtaining definite results on optimal stimulation protocols [8,70]. Low-frequency electrical and optical stimulation (< 5Hz) applied during interictal phases has been shown to reduce the frequency of interictal spikes and seizure initiation in animal and human studies [58,71]. High-frequency electrical stimulation (>100 Hz) applied during ictal phases has also been shown to terminate seizures [72–74]. Here, we considered the pre-ictal phase, which is between the interictal and ictal phases. We showed that stimulation with a frequency greater than 20 Hz can abort pre-ictal oscillations and keep the system close to background activity by depolarizing the subpopulation of SOM+ interneurons. From our results, the suggested frequency range lies between the ranges of low- and high-frequency stimulations and beyond. This can be due to the fact that the considered epileptogenic phase (pre-ictal) is “in-between” the phases where low-frequency (interictal phase) and high-frequency (ictal phase) stimulations are successful. Furthermore, a single-lumped NMM considers the local synchronized activity at the mesoscopicscale, whereas epilepsy is a large-scale network disease where heterogeneity and spatial dynamics can be crucial for epileptic activity. Therefore, spatio-temporal effects of ongoing neural dynamics, synaptic plasticity or differences in the activation functions or neural subpopulations could not be studied in our model. Nevertheless, our study explains the complex locally observed pre-ictal patterns. Furthermore, our results suggest an alternative stimulation protocol in terms of frequency and timing of stimulation delivery.

Overall, our study adds to the recent literature on computational studies of seizure abortion. In the context of absence seizures, Taylor et al. [75] have proposed application of single pulse stimulation in a model known to have bistable properties. While close in the level of description to [75], our model differs in two aspects. First, our model of focal seizures is different than of absence seizure due to the difference between underlying neural circuits. Second, our mathematical analysis is based on the slow-fast features of the dynamical system. Therefore, transitions from epileptic discharges to normal background activity are obtained by stimulations which differ from a single pulse not only in terms of duration, but also in terms of waveform.

It has long been reported that pre-ictal spiking/bursting is an emerging feature of the interictal to ictal transition and is specific to epileptogenic regions. From a mathematical viewpoint, both spiking (a single bump followed by a quiescent phase) and bursting (a sequence of spikes (bumps) followed by a quiescent phase) oscillations in a neural context result from the interaction between the slow and fast variables of a multiple time-scale system. While the type of the oscillation depends on the bifurcation structure of the fast subsystem, it is always the slow subsystem that drives the recurrent transitions between the quiescence and active (spiking) phases [32], here the subpopulation of SOM+ interneurons. Since the essence of spiking/bursting is the same in general sense, stimulation protocols mainly affecting slow oscillations during the pre-ictal phase would abort pre-ictal spiking/bursting activity. In other words, the burst-abortion strategy presented in this paper would also be appropriate to abort spiking. Yet, it is essential to identify the neuronal subpopulations of the brain region under consideration, the connections between these neuronal subpopulations and their roles in such slow-fast regimes to optimize the stimulation frequency, since as shown in this manuscript, subpopulations with slower kinetics are more responsive to pulsed stimulations. For instance, a pre-ictal spiking regime mediated by GABA_B_ interneurons may be aborted by using lower stimulation frequencies than a pre-ictal spiking regime mediated by GABA_A_ interneurons, since GABA_B_ interneurons have slower kinetics than GABA_A_ interneurons. Depending on the neuroanatomy and neurophysiology of a specific brain region (type of subpopulations and connections between them), activation of specific types of interneurons can be achieved *via* the modulation of different neural targets [57,58,60,76–78].

LFPs recorded by SEEG electrodes can provide hints on timescale separations, since there is a tight link between the form of the observed complex oscillations and timescale separations. For instance, the pre-ictal bursts examined in this work involve fast spikes followed by a slower large amplitude discharge. While fast spikes indicate an interaction of fast and slow components, the slower large amplitude discharge spikes indicate an interaction of slow and super-slow components. The natural presence of three distinct timescales in the model, which are introduced by the kinetics of glutamatergic receptors (NMPA and AMPA) and GABAergic receptors of two distinct types, enables modeling these complex oscillations. However, computation of the synaptic time scales from the LFP is an-ill posed problem since the LFP is a complex mixture of excitatory and inhibitory post-synaptic potentials at the level of pyramidal neurons.

Slow-fast analysis of the mathematical models of neural systems with complex oscillatory patterns has contributed to discover the roles of biological ingredients [79–88], unveil the fine structures (e.g. excitability thresholds, spike adding mechanisms and subthreshold oscillations …etc.) [30,35,89–96], and design controllers [97,98]. Slow-fast thinking has also been insightful for constructing phenomenological models of epileptic dynamics and analyzing their dynamics [99–101]. Recently, response types of brief electrical pulses in coupled NMMs have been investigated using some elements of slow-fast analysis [102]. In [103] a regime of canard solutions has been reported in sleep/wake transitions in a NMM, also in an extended NMM formulation in [84]. As opposed to [82–84], here we reformulated a widely studied NMM in an explicit slow-fast form and unveiled its three-time-scale structure. Thus, the system is an example of three-time-scale systems that are beginning to be explored. Besides, the structure of the model is widely used in engineering studies, in particular in systems with feedback [33]. Hence, the methodology used is this paper could be beneficial in many other research areas.

During our investigations we also observed canard solutions organizing the transition from slow-wave (≈6 Hz) to bursting oscillations through a spike-adding mechanism in between. We did not further explore this interesting mechanism since the main purpose of this paper was to understand the perturbation effect on pre-ictal bursting solutions, which are away from the canard regime in the parameter space. Further analysis concerning the classification of slow dynamics near the fold points, canard solutions and spike-adding mechanisms in the line of [36,37,104] are among the possible extensions of this work.

Another possible avenue to extend this work would be to consider the possibility to perform patient-specific bifurcation analyses of epileptiform patterns to propose patient-specific stimulation parameters (most critically, stimulation frequency) that would result in the abortion of the said epileptiform patterns. Current direct brain stimulation protocols in epilepsy use indeed relatively generic parameters, without consideration for the type, localization or extent of the epileptogenic network; a possible factor to explain the lack of consistency for this therapy so far for drug-refractory epilepsy. For our methods to be applicable an adaptive closed-loop detection system, such as a brain responsive neurostimulation system, can be taken into account, which could detect pre-ictal discharges, and then intervene with an appropriate stimulation.

Finally, we should emphasize that the NMM considered here was initially proposed a model for hippocampal activity. As shown in this study, this NMM can reproduce complex oscillatory patterns at the macroscopic level resulting from interaction of district subpopulations with different kinetics. More recently, the model was shown to have a more general scope as the embedded circuitry is valid that of most of the regions at macroscopic level (see [105] and references there in). Thus, appropriately formulated NMMs and the tools presented here can be used to study the complex dynamics observed in other cortical areas and to investigate effects of external perturbations.

## Supporting information

S1 Fig Block diagram of the neural mass model (1). The model features three types of neuronal subpopulations, namely pyramidal neurons (PYR and PYR’), GABAergic SOM+ interneurons (SOM+) and GABAergic PV+ interneurons (PV+). Average PSP at the level of each subpopulation (denoted by *y* variables) is determined by a pulse-to-wave function *S*(*v*) and a linear dynamic transfer function *h*(*t*). Properties of *h*(*t*) are determined by synaptic gains (*A*, *B*, *G*) and synaptic time constants (1/*a*, 1/*b*, 1/*g*). Parameters *C*_*i*_s denote average synaptic contacts. Cortical input is denoted by *p*(*t*).
(TIF)

S2 Fig Periodic orbits of (4) and (8) for ε = 0.01. The system is put in the bursting regime by taking B = 18 and the other parameters are as given in Table 1. (a) Solution of (3) for ε = 0.01 for projected on the bifurcation diagram (black curve) of (4) for ε = 0 where *v*_2_ is treated as a parameter. Stable and unstable solutions are indicated with bold and dashed lines, respectively. The equilibrium points along the black Z-shaped curve are unstable on the middle branch of the curve, between the limit points (LP) *LP*_1_ and *LP*_2_ (black dots), and on the upper branch between the supercritical Hopf (H) bifurcation points *H*_1_ and *H*_2_ (green dots). The amplitude of the stable limit cycles is bounded by the green continuous curves connecting the *H*_1_ and *H*_2_ points in the ε = 0 limit. Arrows show the direction of the flow. (b) Solution of (7) for ε = 0.01 projected on the bifurcation diagram of (8) (red curve) where *v*_2_ is treated as a parameter. Stable and unstable solutions are indicated with bold and dashed lines, respectively. The equilibrium points along the black Z-shaped curve are unstable on the middle branch of the curve, between the *LP*_1_ and *LP*_2_ limit points (red dots). Arrows show the direction of the flow.
(TIF)

S3 FigResponse of a neural mass block to biphasic balanced pulses.A neural mass (NM) block, that is $\ddot{y}=M/\tau S(I_{ext}(t))-2/\tau \dot{y}-{1/\tau }^{2}y$ with *τ* = {0.05, 0.01, 0.003} and *Mτ* = 1, receives biphasic pulses *I*_*ext*_(*t*) at different pulse frequency, amplitude and width. Although *Mτ* is constant across the trials, the amplitude of the response varies due to the difference between the synaptic kinetics. (a) Amplitude of the steady state oscillations of NM evoked by *I*_*ext*_(*t*) of pulse width 0.05 ms, amplitude 1 (arb. unit) and frequency in *f* = [1, 100]*Hz*. Red dashed line is the base level (denoted by *S*(0)) of the NM in the absence of any inputs. The NM with slow kinetics (*τ* = 0.05) detaches from the base level for a smaller stimulation frequency than the NM with fast kinetics (*τ* = 0.003).) Amplitude of the steady state oscillations decreases with frequency. Panels (a1) and (a2) show the responses at *f* = 10 *Hz* and *f* = 100 *Hz*, respectively. Same color codes are used in panels (a), (a1) and (a2). (b) Amplitude of the steady state oscillations of NM evoked by *I*_*ext*_(*t*) of pulse width 0.05 ms, frequency *f* = 10 *Hz* and amplitude *amp* = [1,100] (*arb*.*unit*). Red dashed line is the base level (denoted by *S*(0)) of the NM in the absence of any inputs. The difference between the base line and min (*y*(*t*)) is larger for the NM with slow kinetics (*τ* = 0.05) than the NM with fast kinetics (*τ* = 0.003).) Amplitude of the steady state oscillations increases with amplitude then does not change further for *amp*>20 (*arb*.*unit*). Panels (b1) and (b2) show the time course of the responses at *amp* = 1 (*arb*.*unit*) and *amp* = 20 (*arb*.*unit*), respectively. Same color codes are used in panels (b), (b1) and (b2). (c) Amplitude of the steady state oscillations of NM evoked by *I*_*ext*_(*t*) of frequency *f* = 10 *Hz*, pulse width *width* = [0.05, 50] ms with amplitude *amp* = 0.05 *width*^−1^(*arb*.*unit*). Red dashed line is the base level (denoted by *S*(0)) of the NM in the absence of any inputs. The NM responds with a lower shoot to increasing the pulse width which appears for narrower pulses for the NM with fast kinetics (*τ* = 0.003) than the NM with slow kinetics (*τ* = 0.03).) Amplitude of the steady state oscillations increases with amplitude then decreases with pulse width. Panels (c1) and (c2) show the time course of the responses at *width* = 10 *ms* and *width* = 50 *ms*, respectively. Same color codes are used in panels (c), (c1) and (c2).(TIF)
