# Supplementary figures and images for: Neural mass modeling of slow-fast dynamics of seizure initiation and abortion
Source: PLoS Comput Biol. 2020 Nov 9;16(11):e1008430. doi: 10.1371/journal.pcbi.1008430 (PMC7676664; doi:10.1371/journal.pcbi.1008430)

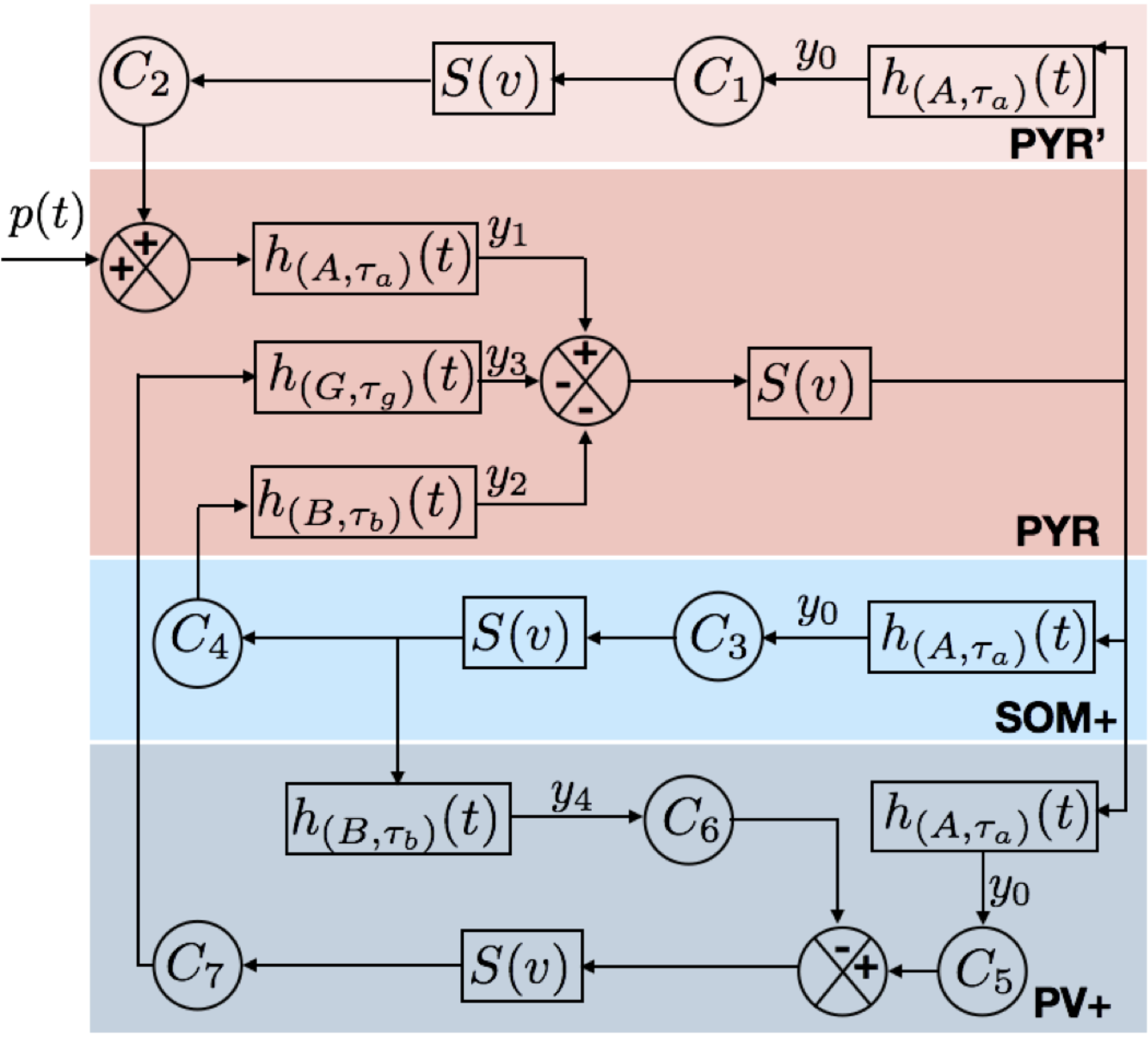

Supplement: S1 Fig — The model features three types of neuronal subpopulations, namely pyramidal neurons (PYR and PYR’), GABAergic SOM+ interneurons (SOM+) and GABAergic PV+ interneurons (PV+). Average PSP at the level of each subpopulation (denoted by y variables) is determined by a pulse-to-wave function S(v) and a linear dynamic transfer function h(t). Properties of h(t) are determined by synaptic gains (A, B, G) and synaptic time constants (1/a, 1/b, 1/g). Parameters Cis denote average synaptic contacts. Cortical input is denoted by p(t). (TIF) [file pcbi.1008430.s001.tif]

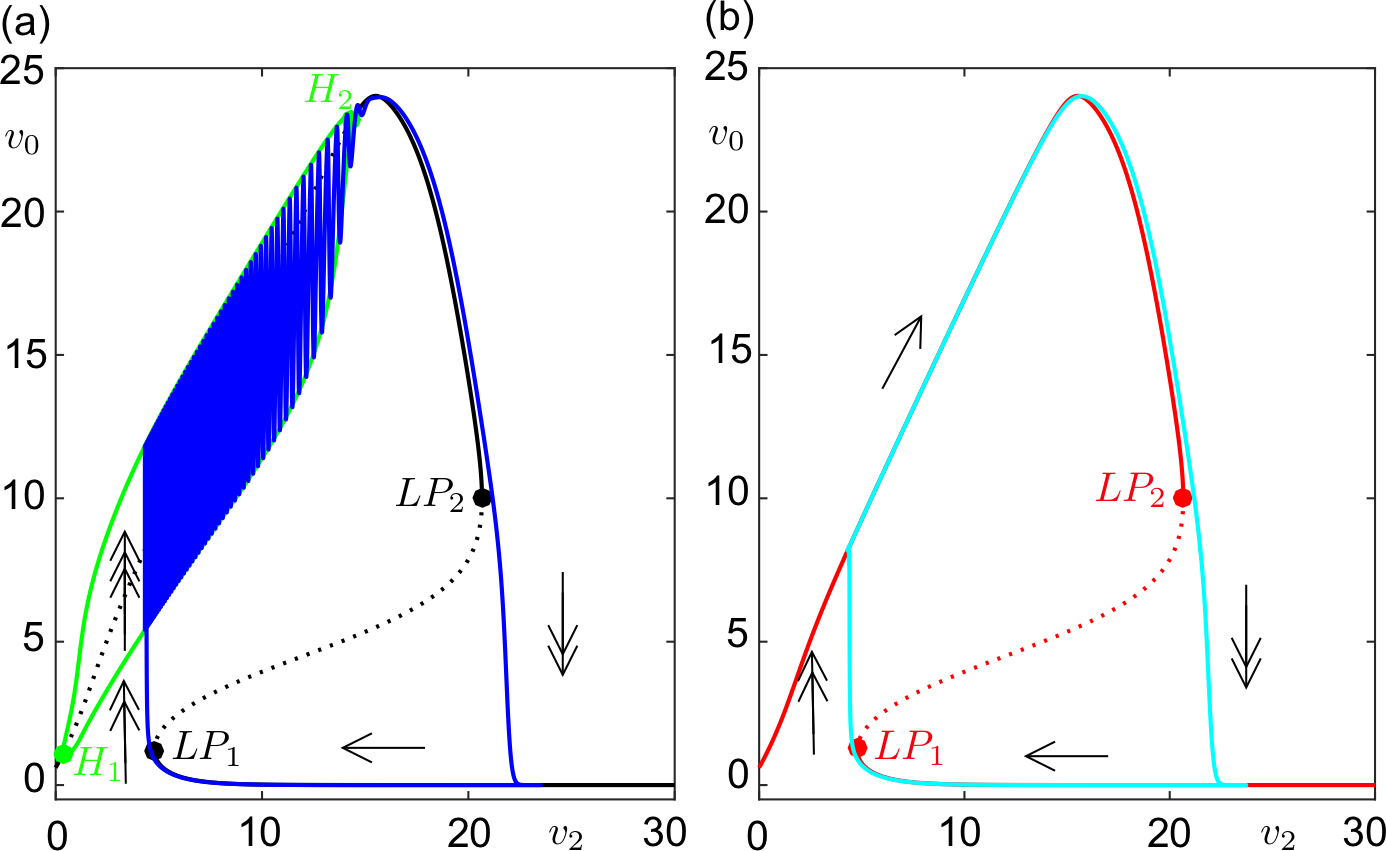

Supplement: S2 Fig — The system is put in the bursting regime by taking B = 18 and the other parameters are as given in Table 1. (a) Solution of (3) for ε = 0.01 for projected on the bifurcation diagram (black curve) of (4) for ε = 0 where v2 is treated as a parameter. Stable and unstable solutions are indicated with bold and dashed lines, respectively. The equilibrium points along the black Z-shaped curve are unstable on the middle branch of the curve, between the limit points (LP) LP1 and LP2 (black dots), and on the upper branch between the supercritical Hopf (H) bifurcation points H1 and H2 (green dots). The amplitude of the stable limit cycles is bounded by the green continuous curves connecting the H1 and H2 points in the ε = 0 limit. Arrows show the direction of the flow. (b) Solution of (7) for ε = 0.01 projected on the bifurcation diagram of (8) (red curve) where v2 is treated as a parameter. Stable and unstable solutions are indicated with bold and dashed lines, respectively. The equilibrium points along the black Z-shaped curve are unstable on the middle branch of the curve, between the LP1 and LP2 limit points (red dots). Arrows show the direction of the flow. (TIF) [file pcbi.1008430.s002.tif]

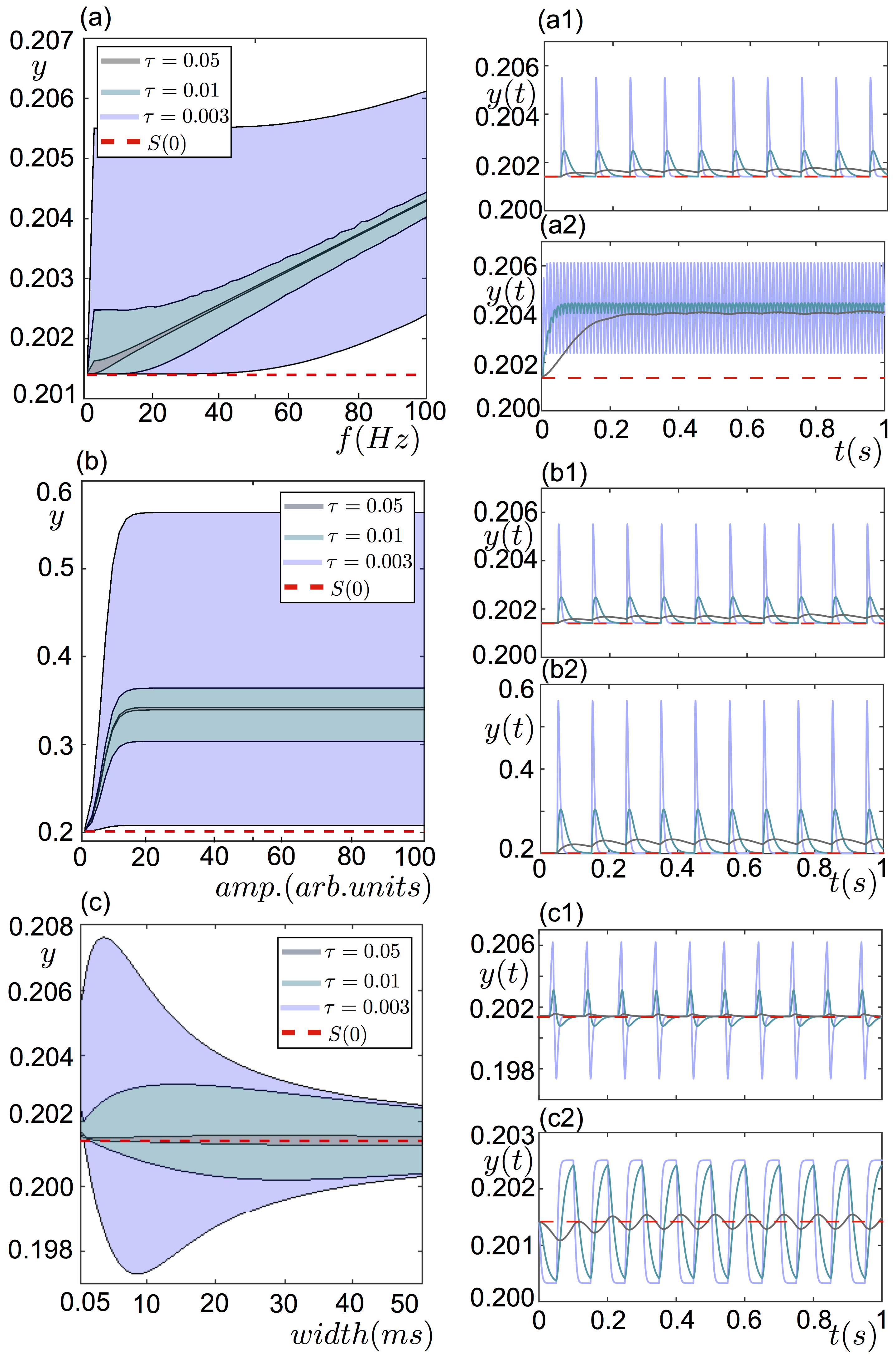

Supplement: S3 Fig — A neural mass (NM) block, that is y¨=M/τS(Iext(t))−2/τy˙−1/τ2y with τ = {0.05, 0.01, 0.003} and Mτ = 1, receives biphasic pulses Iext(t) at different pulse frequency, amplitude and width. Although Mτ is constant across the trials, the amplitude of the response varies due to the difference between the synaptic kinetics. (a) Amplitude of the steady state oscillations of NM evoked by Iext(t) of pulse width 0.05 ms, amplitude 1 (arb. unit) and frequency in f = [1, 100]Hz. Red dashed line is the base level (denoted by S(0)) of the NM in the absence of any inputs. The NM with slow kinetics (τ = 0.05) detaches from the base level for a smaller stimulation frequency than the NM with fast kinetics (τ = 0.003).) Amplitude of the steady state oscillations decreases with frequency. Panels (a1) and (a2) show the responses at f = 10 Hz and f = 100 Hz, respectively. Same color codes are used in panels (a), (a1) and (a2). (b) Amplitude of the steady state oscillations of NM evoked by Iext(t) of pulse width 0.05 ms, frequency f = 10 Hz and amplitude amp = [1,100] (arb.unit). Red dashed line is the base level (denoted by S(0)) of the NM in the absence of any inputs. The difference between the base line and min (y(t)) is larger for the NM with slow kinetics (τ = 0.05) than the NM with fast kinetics (τ = 0.003).) Amplitude of the steady state oscillations increases with amplitude then does not change further for amp>20 (arb.unit). Panels (b1) and (b2) show the time course of the responses at amp = 1 (arb.unit) and amp = 20 (arb.unit), respectively. Same color codes are used in panels (b), (b1) and (b2). (c) Amplitude of the steady state oscillations of NM evoked by Iext(t) of frequency f = 10 Hz, pulse width width = [0.05, 50] ms with amplitude amp = 0.05 width−1(arb.unit). Red dashed line is the base level (denoted by S(0)) of the NM in the absence of any inputs. The NM responds with a lower shoot to increasing the pulse width which appears for narrower pulses for the NM with fast kin [file pcbi.1008430.s003.tif]
